# Hyphopodium-Specific VdNoxB/VdPls1-Dependent ROS-Ca^2+^ Signaling Is Required for Plant Infection by *Verticillium dahliae*

**DOI:** 10.1371/journal.ppat.1005793

**Published:** 2016-07-27

**Authors:** Yun-Long Zhao, Ting-Ting Zhou, Hui-Shan Guo

**Affiliations:** 1 State Key Laboratory of Plant Genomics and National Center for Plant Gene Research (Beijing), Institute of Microbiology, Beijing, China; 2 University of the Chinese Academy of Sciences, Beijing, China; University of Nebraska-Lincoln, UNITED STATES

## Abstract

*Verticillium dahliae* is a phytopathogenic fungus obligate in root infection. A few hyphopodia differentiate from large numbers of hyphae after conidia germination on the root surface for further infection. However, the molecular features and role of hyphopodia in the pathogenicity of *V*. *dahliae* remain elusive. In this study, we found that the VdPls1, a tetraspanin, and the VdNoxB, a catalytic subunit of membrane-bound NADPH oxidases for reactive oxygen species (ROS) production, were specifically expressed in hyphopodia. VdPls1 and VdNoxB highly co-localize with the plasma membrane at the base of hyphopodia, where ROS and penetration pegs are generated. Mutant strains, Vd*Δnoxb* and Vd*Δpls1*, in which *VdPls1* and *VdNoxB* were deleted, respectively, developed defective hyphpodia incapable of producing ROS and penetration pegs. Defective plasma membrane localization of VdNoxB in Vd*Δpls1* demonstrates that VdPls1 functions as an adaptor protein for the recruitment and activation of the VdNoxB. Furthermore, in Vd*Δnoxb* and Vd*Δpls1*, tip-high Ca^2+^ accumulation was impaired in hyphopodia, but not in vegetative hyphal tips. Moreover, nuclear targeting of VdCrz1 and activation of calcineurin-Crz1 signaling upon hyphopodium induction in wild-type *V*. *dahliae* was impaired in both knockout mutants, indicating that VdPls1/VdNoxB-dependent ROS was specifically required for tip-high Ca^2+^ elevation in hyphopodia to activate the transcription factor *VdCrz1* in the regulation of penetration peg formation. Together with the loss of virulence of Vd*Δnoxb* and Vd*Δpls1*, which are unable to initiate colonization in cotton plants, our data demonstrate that VdNoxB/VdPls1-mediated ROS production activates VdCrz1 signaling through Ca^2+^ elevation in hyphopodia, infectious structures of *V*. *dahliae*, to regulate penetration peg formation during the initial colonization of cotton roots.

## Author Summary

Beyond the harmful roles in pathological processes and aging, growing evidence indicates that reactive oxygen species (ROS) play a pivotal role in physiological processes, including cell differentiation and development in fungi. *Verticillium dahliae* Kleb., a soil-born phytopathogenic fungus, germinates larger numbers of hyphae on root surface, a few of which differentiate into hyphopodia during initial colonization. In this study, we verified that a specific membrane-bound NADPH oxidase complex (Nox), composed of the catalytic subunit VdNoxB and the tetraspaninVdPls1, is responsible for the ROS burst and penetration peg differentiation within the hyphopodium, which is essential for redirecting fungal growth toward host cells to penetrate cotton roots and to colonize the host vascular system. Moreover, we found that VdPls1/VdNoxB-mediated ROS production elevates tip-high cytoplasmic free calcium ions (Ca^2+^) in hyphopodia to activate conservation calcineurin-Crz1 signaling for the establishment polarized penetration peg formation. In plant root-hair cells, endogenous ROS elevation activates tip-high Ca^2+^ for root-hair cell polarity and elongation. Taken together, we propose that Ca^2+^, as a second messenger for transduction of the apical ROS signal, is a hallmark and common regulatory mechanism for apical growth in eukaryotes.

## Introduction

*Verticillium dahliae* Kleb. is a phytopathogenic fungus that causes wilt disease in a wide range of crops, including cotton[1]. Infection of cotton roots by *V*. *dahliae* in soil leads to the colonization of vascular tissues in host plants[2,3]. By generating a reporter *V*. *dahliae* strain expressing GFP (V592-GFP) for the infection of *Arabidopsis thaliana* roots, we recently showed that fungal germ tube emergence from the infected root surface following inoculation with spores from V592-GFP extends longitudinally along root epidermal cells. Subsequently, a few swollen hyphae were observed, followed by the formation of a narrow penetration peg during perforation of the junctions of epidermal cells of roots[4]. Pathogens have evolved different strategies to overcome the various barriers that they encounter during infection of their hosts. For example, the rice blast fungus *Magnaporthe oryzae* develops penetration structures known as appressoria, which are heavily melanized when they are inoculated on plant leaves. Interestingly, under laboratory conditions, *M*. *oryzae* is able to colonize roots, but the process is mediated by simple penetration structures comprised of swollen hyphae known as hyphopodia, instead of the melanized appressoria[5,6]. The swollen hyphae of *V*. *dahliae* were also suggested to be hyphopodia[7]. However, it is not known whether there is a specific molecular feature in hyphopodia that is distinct from vegetative hyphae of *V*. *dahliae*. Neither the regulation of penetration pegs formed from hyphopodia nor their role in plant infection have been investigated.

The differentiation of a penetration peg from an appressorium of fungi, such as the *M*. *oryzae*, requires generation of reactive oxygen species (ROS)[8,9,10]. The major enzymatic producer of ROS are membrane-bound NADPH oxidases (Nox), a ubiquitous group of eukaryotic flavoenzymes that catalyze the reduction of dioxygen to the superoxide anion using electrons provided by NADPH[11,12,13]. Nox enzymes have been intensively studied, especially in mammalian cells. The most well studied member is the mammalian Nox2 complex, which is responsible for the oxidative burst in phagocytes in response to microbial infection[11]. An integral membrane flavocytochrome heterodimer, composed of the catalytic subunit gh91^*phox*^ and the adaptor protein p22^*phox*^, is essential for catalytic activity of the Nox2 complex. Two homologues of the mammalian gp91^*phox*^, NoxA (Nox1) and NoxB (Nox2), have been identified in fungi[14,15] and have been shown to be crucial for distinct fungal cellular differentiation during sexual reproduction and developmental processes that involve transitions from non-polarized to polarized cell growth, such as hyphal tip growth and tissue invasion by fungi, and fungal virulence[8,9,15,16,17,18,19,20,21,22,23,24,25]. Recently, a functional homologue of the mammalian adaptor protein p22^*phox*^, named NoxD, was identified in *Podospora anserina*[26] and *Botrytis cinerea*[27]. Direct physical interaction and similar functions between BcNoxA and BcNoxD in differentiation and pathogenicity suggest that NoxA/NoxD is the fungal equivalent of the mammalian gp91^*phox*^/p22^*phox*^ flavocytochrome complex[13,26,27]. However, a fungal homologue of the mammalian p22^*phox*^ for the catalytic subunit NoxB has not been identified. The tetraspanin (Pls1) homologues of several fungal pathogens were found to be expressed during appressoria development. Overlapping phenotypes of Pls1 and NoxB deletion mutants of *P*. *anserina* and *B*. *cinerea* have been shown [28,29], and it was suggested that Pls1 is the corresponding integral membrane adaptor for the assembly of the NoxB complex. However, direct evidence of Pls1 as the adaptor protein for NoxB is still lacking.

As signaling molecule regulators, ROS play pivotal roles in the control of tip growth, which is a common mode of cell expansion and morphogenesis in eukaryotic kingdoms[30]. In plant root-hair cells, endogenous ROS elevation produced by the plasma membrane NADPH oxidase has been shown to activate hyperpolarization-activated calcium ion (Ca^2+^) channels to facilitate Ca^2+^ influx for root-hair cell polarity and elongation[31,32]. As in root-hair cells, the typical tip growth intracellular organization in fungal hyphae is also highly polarized and contains elevated tip-focused Ca^2+^ levels[32,33]. However, it is not known whether ROS produced by the membrane-bound NADPH oxidases coupled with Ca^2+^ elevation have a specific effect on polarized penetration peg formation. Moreover, in addition to ROS-Ca^2+^ in morphogenesis, regulatory role of ROS-Ca^2+^ in fungal pathogenicity remains unknown.

The objectives of this study were (i) to verify that VdPls1 is the corresponding integral membrane adaptor for the assembly and activation of the NoxB in *V*. *dahliae*, by demonstrating the co-localization of VdPls1 and VdNoxB, and the requirement of VdPls1 for plasma membrane localization of VdNoxB in hyphopodia; (ii) to determine if VdNoxB/VdPls1-produced ROS are required for penetration peg morphogenesis; and (iii) to determine if VdNoxB/VdPls1-produced ROS are required for the establishment of Ca^2+^-mediated penetration peg formation, and the resulting effect on pathogenicity by characterization of the Ca^2+^-dependent transcriptional factor VdCrz1 and relative genes in response to hyphopodium induction. Our data establish the molecular features and functions of hyphopodia in the pathogenicity of *V*. *dahliae*.

## Result

### VdNoxB and VdPls1 are required in the initial colonization of *V*. *dahliae* on cotton plants

To investigate the functions of *VdNoxB* and *VdPls1* in the development and pathogenicity of *V*. *dahliae*, the *VdNoxB* and *VdPls1* gene sequences were first amplified from *V*. *dahliae* V592 isolate from cotton, based on a tblastn search using *M*. *oryzae* Nox2 or Pls1 and the database for VdLs.17, a *V*. *dahliae* isolate from lettuce[34]. Knockout mutants, Vd*Δnoxb* and Vd*Δpls1*, were generated using the homologous recombination method[35]. A single copy of *VdNoxB* and *VdPls1* in V592 was deleted (S1 Fig), and the expression of each mRNA was abolished in Vd*Δnoxb* and Vd*Δpls1* mutants (S1 Fig).

**Fig 1:**
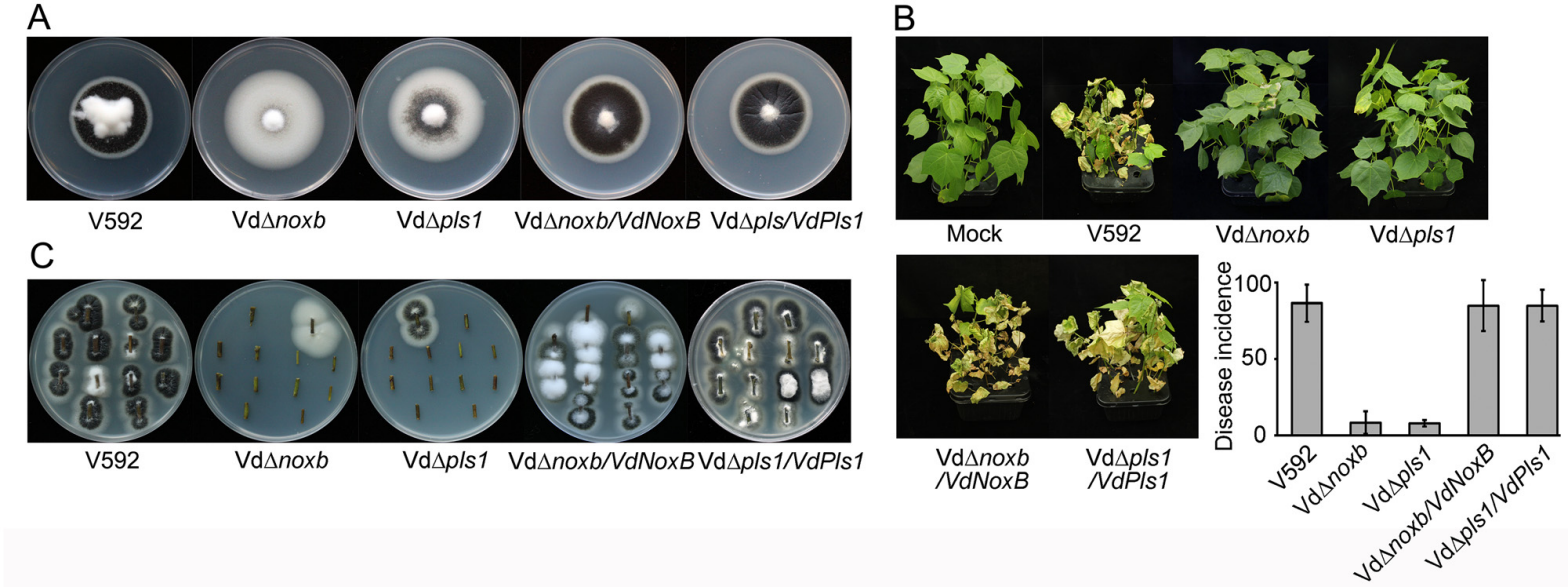
Colony morphology and virulence of Vd*Δnoxb* and Vd*Δpls1*. **A.** Colony morphology of wild-type V592, Vd*Δnoxb* and Vd*Δpls1* mutant strains, and Vd*Δnoxb*/*VdNoxB* and Vd*Δpls1*/*VdPls1* complemented strains on PDA plates after 2 weeks post-incubation. Both mutants show rapid growth and dramatically reduced melanin production, and complemented strains restore to the wild-type morphology. **B.** Disease symptoms of cotton plants infected with V592, mutant and complemented strains at 30 days post-incubation (dpi). The incidence of disease was evaluated with three replicates of 36 plants, and standard deviations are indicated by the error bars. **C.** Stem sections from inoculated cotton plants on PDA plates. Each section was from a different plant.

The Vd*Δnoxb* and Vd*Δpls1* mutant colonies exhibited higher hyphal growth rates and dramatically reduced melanin production on potato dextrose agar (PDA) plates compared to wild-type V592 (Fig 1A). Two-week-old cotton seedlings were inoculated with spores from the Vd*Δnoxb*, Vd*Δpls1* mutant or wild-type V592 strains using the unimpaired root dip-inoculation method[36]. The aggressiveness of disease, with leaf wilt, defoliation and dried whole seedlings at 30 day-post-inoculation (dpi) was observed for most seedlings inoculated with V592 (Fig 1B). Both Vd*Δnoxb* and Vd*Δpls1* mutant displayed significantly reduced disease severity in cotton (Fig 1B). Correspondingly, fungal hyphae were recovered from the V592-infected cotton plants, but rarely from either knockout mutant-infected plants (Fig 1C). Both colony morphologies and pathogenicity were restored in either Vd*Δnoxb*/*VdNoxB* or Vd*Δpls1*/*VdPls1* complemented strains (Fig 1). These data demonstrate targeted disruption of *VdNoxB* and *VdPls1* and suggest that the loss of virulence of *V*. *dahliae* mutants knocked out for either *VdNoxB* or *VdPls1* was due to defects in the colonization in cotton plants.

### *VdNoxB* and *VdPls1* are indispensable for penetration peg formation

**Fig 2:**
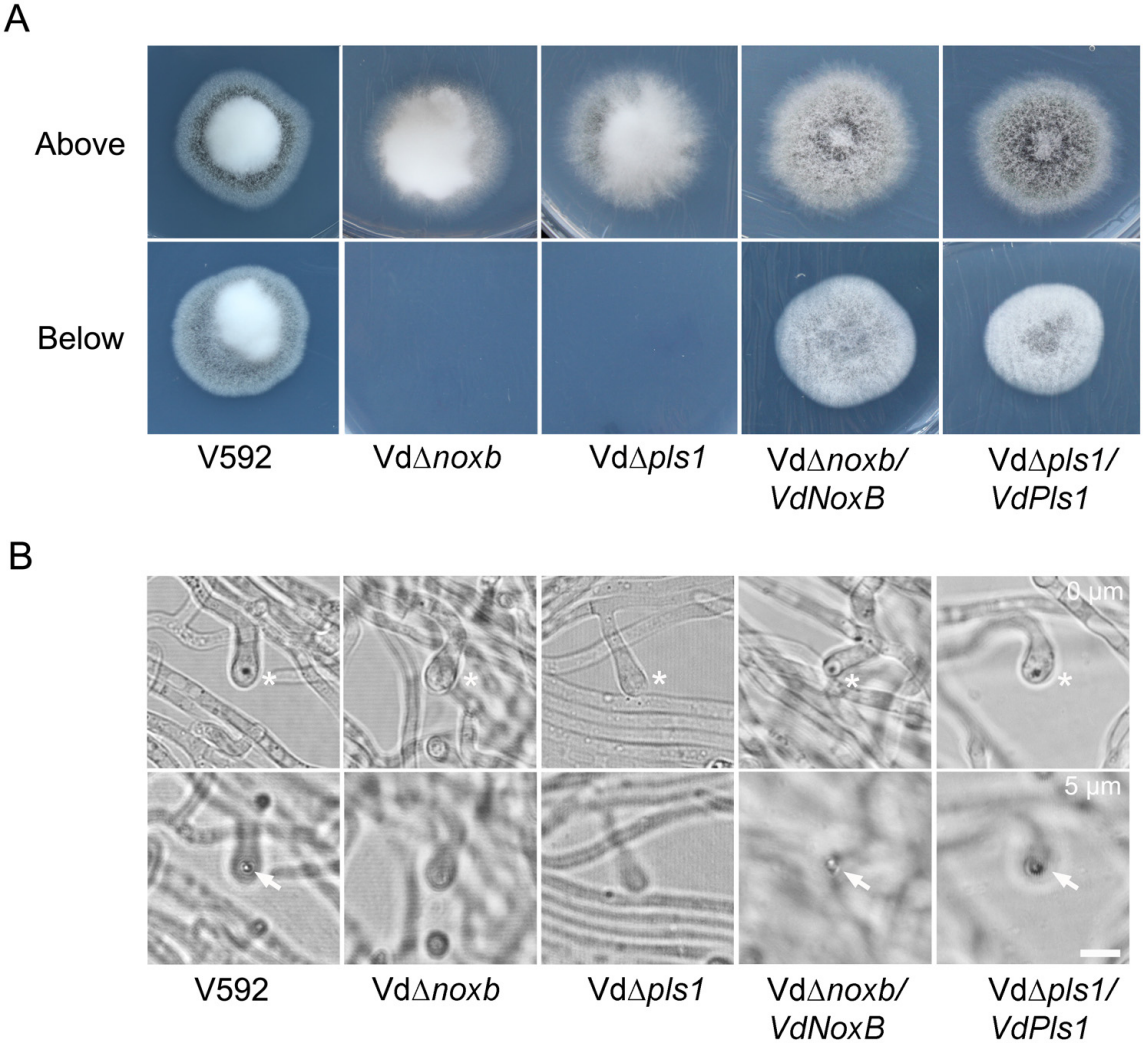
Penetration assay on the cellophane membrane. **A.** Colonies of V592, Vd*Δnoxb* and Vd*Δpls1* mutant strains, Vd*Δnoxb*/*VdNoxB* and Vd*Δpls1*/*VdPls1* complemented strains grown on MM medium overlaid with a cellophane layer (above) and removal of the cellophane membrane (below). Photographs in the first row were taken at 7 dpi. The second row shows growth of a V592 colony on MM medium after penetration from the cellophane membrane; neither mutant strain breached the cellophane membrane to grow on medium. Complemented strains restore the penetration ability. **B.** Observation of penetration peg development on the cellophane membrane at 2 dpi. Differentiation of hyphopodia (swollen hyphae) in V592, mutant and complemented strains was indicated by asterisks in the first row. Penetration pegs (the dark pin) were only observed in V592 and complemented strains from the hyphopodium indicated by the arrow in the second row, which was focused at 5μm below the first row. Bar = 5μm.

To investigate the roles of *VdNoxB* and *VdPls1* during the initial colonization of *V*. *dahliae*, the penetration abilities of Vd*Δnoxb* and Vd*Δpls1* were first examined by incubation of the mutant strains, wild-type V592 and complemented strains, Vd*Δnoxb*/*VdNoxB* and Vd*Δpls1*/*VdPls1*, on a cellophane membrane laid on minimal medium, which is used for appressoria induction in *M*. *grisea*[37]. At 3 dpi, fungal hyphae penetration from the cellophane membrane and growth on medium was observed for V592 and both complemented strains when the cellophane membrane was removed (Fig 2A). Neither the Vd*Δnoxb* nor Vd*Δpls1* mutant hyphae penetrated the cellophane membrane, even though the cellophane membrane was not removed until 7 dpi (Fig 2A). We then observed hyphae under microscopy. V592 and complemented strains developed swollen hyphae, appressorium-like hyphopodia, with clear penetration pegs to breach the membrane. However, both Vd*Δnoxb* and Vd*Δpls1* mutants produced defective hyphopodia without penetration peg formation (Fig 2B).

**Fig 3:**
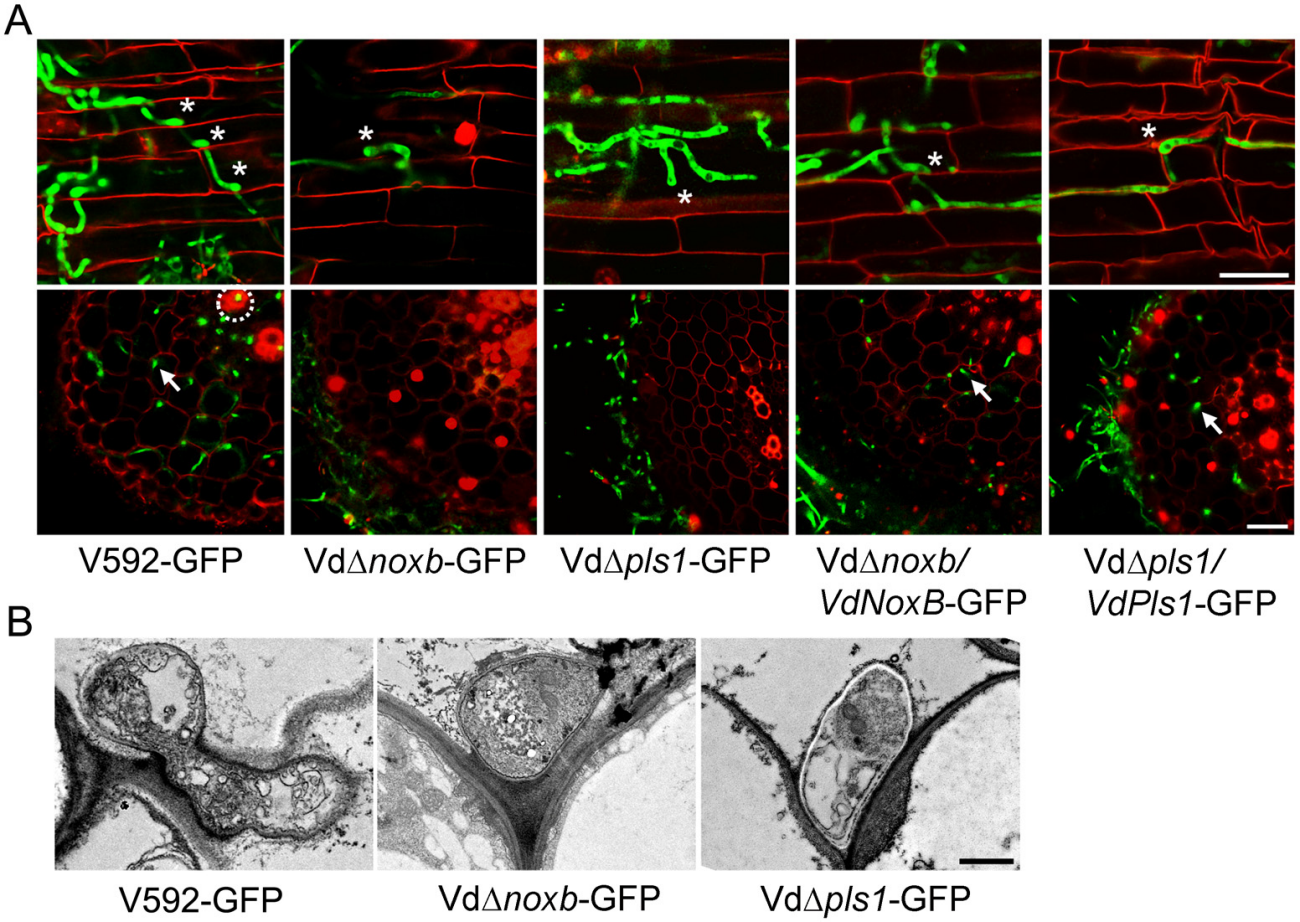
Observation of fungal penetration in cotton roots. **A.** Confocal laser scanning microscopy (CLSM) images of hyphopodium development and/or penetration of V592-GFP, mutant strains Vd*Δnoxb-*GFP and Vd*Δpls1-*GFP and complemented strains Vd*Δnoxb*/*VdNoxB*-GFP and Vd*Δpls1*/*VdPls*-GFP. Photographs were taken on the surface (first row) or the cross section (second row) of cotton root at 3dpi and 5dpi, respectively. Asterisks indicate the hyphopodium. Root xylem structure was labeled by white dotted line, and invasive hyphae were indicated with arrows. Bar = 25 μm. **B.** Transmission electron microscope (TEM) images show penetration of a V592-GFP hyphopodium, but not those of the Vd*Δnoxb*-GFP or Vd*Δpls1*-GFP mutants, through the root cell wall. Bar = 1 μm.

To further confirm the penetration defect on root colonization, the reporter *GFP* gene was transformed to the mutant and complemented strains to produce Vd*Δnoxb-*GFP, Vd*Δpls1-*GFP, Vd*Δnoxb*/*VdNoxB*-GFP and Vd*Δpls1*/*VdPls*-GFP. Confocal laser scanning microscopy (CLSM) observation shows that wild-type V592-GFP and complemented strains Vd*Δnoxb*/*VdNoxB*-GFP and Vd*Δpls1*/*VdPls*-GFP colonized on the root surface and developed hyphopodia at 3 dpi (Fig 3A), followed by perforation of the junctions of epidermal cells. Then invasive hyphae crossed the root cortical cells, reached the vascular cylinder and colonized the xylem vessels at 5 dpi (Fig 3A). In contrast, both Vd*Δnoxb-*GFP and Vd*Δpls1-*GFP mutants developed hyphopodia on the root surface at 3 dpi that were incapable of further perforation of epidermal cells, and the mutant hyphae kept growing on the rhizodermis at 5 dpi (Fig 3A). Consistent with this, transmission electron microscopy (TEM) showed a wild-type V592 hyphopodium followed by an invasive hyphae that breached the root cell wall (Fig 3B), whereas both mutants failed to produce penetration pegs from their hyphopodia (Fig 3B). These results indicate that *VdNoxB* and *VdPls1* are individually indispensable for penetration peg formation in *V*. *dahliae*.

### Co-localization of VdNoxB and VdPls1 in hyphopodia

**Fig 4:**
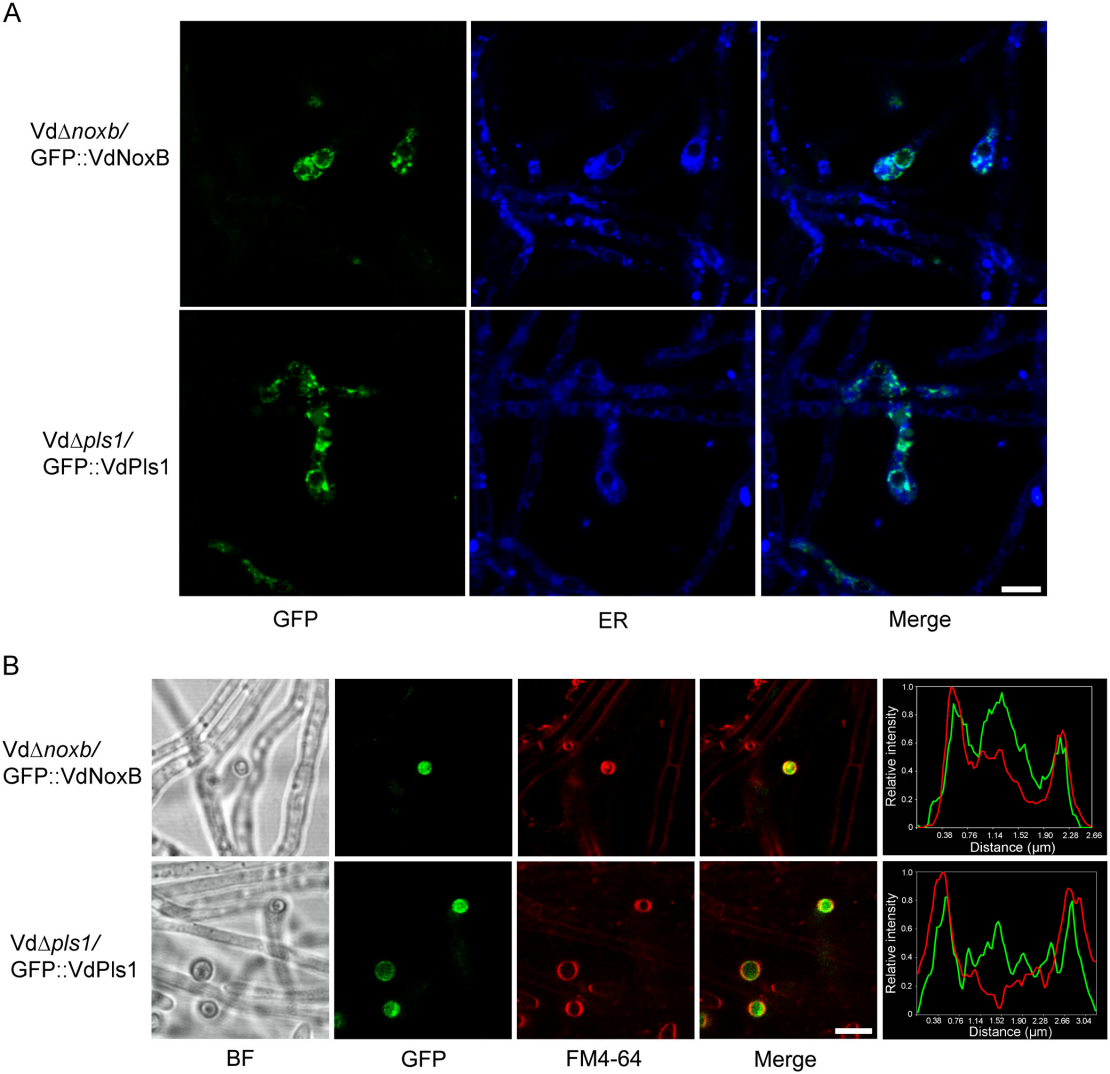
CLSM observation of cellular localization of VdNoxB and VdPls1. **A.** Localization of GFP::VdNoxB and GFP::VdPls1 in hyphopodia at small vesicles and the ER. Vd*Δnoxb*-GFP::VdNoxB and Vd*Δpls1*-GFP::VdPls1 strains were inoculated on MM medium overlaid with cellophane membrane. Photographs were taken at 2 dpi. ER was stained by ER-Tracker Blue-White DPX. **B.** CLSM observation focused at the base of hyphopodium and corresponding linescan graphs. Co-localization of either GFP::VdNoxB- or GFP::VdPls1 with plasma membrane stained with FM4-64. Linescan graphs showing either VdNoxB or VdPls1 localizes on the plasma membrane in a transverse section of individual hyphopodium. Bar = 5 μm.

Identical functions of *VdNoxB* and *VdPls1* in pathogenicity and penetration peg formation suggest that VdPls1 is very likely in the NoxB complex in *V*. *dahliae*. To further confirm this, the cellular localization of VdNoxB and VdPls1 was first examined. GFP was fused at the N-terminus of VdNoxB or VdPls1 to produce GFP::VdNoxB and GFP::VdPls1 under either native promoter and introduced into Vd*Δnoxb* or Vd*Δpls1* mutants. The functional activities of GFP::VdNoxB and GFP::VdPls1 were confirmed by complementation of both penetration ability and pathogenicity in either mutant strain (S2A–S2D Fig). Cellular localization of two fusion proteins was examined under CLSM. Similar cellular localization of GFP::VdNoxB and GFP::VdPls1 was observed in hyphae grown on the cellophane membrane: both fusion proteins were specifically expressed in the hyphopodium at vesicle-like organelles and associated with the ER, which was stained by ER-Tracker Blue-White DPX (Fig 4A), consistent with previous observation[6,29]. Notably, intensive GFP signals were derived from vesicle aggregation and mostly co-localized with the membrane (stained with FM4-64) at the base of hyphopodia, where the penetration pegs developed (Fig 4B).

We then examined the interaction between VdNoxB and VdPls1 by yeast two-hybrid (Y2H) and bimolecular fluorescence complementation (BiFC) assays. For Y2H assay based on split ubiquitin system, the cDNA sequences of *VdNoxB* and *VdPls1* were inserted to pPR3-N and pBT3-N, respectively. *In vitro* direct physical interaction between VdNoxB and VdPls1 was evidenced (S3A Fig). *In vivo* interaction was further examined by BiFC. The C- and N-terminal fragments of Venus were fused upstream of *VdNoxB* and *VdPls1*, respectively, under either native promoter, yielding VC::VdNoxB and VN::VdPls1 for V592 co-transformation. Most of the florescent signal was detected in small vesicles and overlapped with the plasma membrane at the base of hyphopodium (S3B Fig). For the control assay, the membrane protein VdMsb2, a homolog in *M*. *oryzae* that has been reported to be essential for appressorium formation, was fused with C-terminal fragment of Venus at the C-terminal end under the oliC promoter and co-expressed with VN::VdPls1 in V592. No florescent signal was observed in either vegetative hyphae or hyphopodia (S3B Fig), where expression of fusion VdMsb2::GFP was detected (S3C Fig). These results suggest possible interaction of VdNoxB and VdPls1 in hyphopodia. To further confirm interaction between two proteins, we generated epitope-tagged VdNoxB- and VdPls1-expressing hyphae by transforming GFP-VdNoxB and 3Flag-VdPls1 plasmids into V592. The expression of the VdNoxB and VdPls1 was verified by immunoblot analysis. Anti-GFP and anti-Flag antibodies were used to detect proteins in the Flag or GFP immunoprecipitation products (S3D Fig). There was no signal detected for either fusion proteins in mutual co-immunoprecipitation assays through exhaustion of effort (S3D Fig). One possible explanation for the failure might be due to the low amount of proteins since they were specifically expressed in the few hyphopodia. Nevertheless, our data demonstrate that co-localization of VdNoxB and VdPls1 in the hyphopodium at small vesicles, and particularly at the position of penetration peg emergence, are consistent with their essential functions for penetration peg formation and the initial colonization of *V*. *dahliae* on cotton plants.

### VdPls1 is essential for the plasma membrane localization and activation of VdNoxB

**Fig 5:**
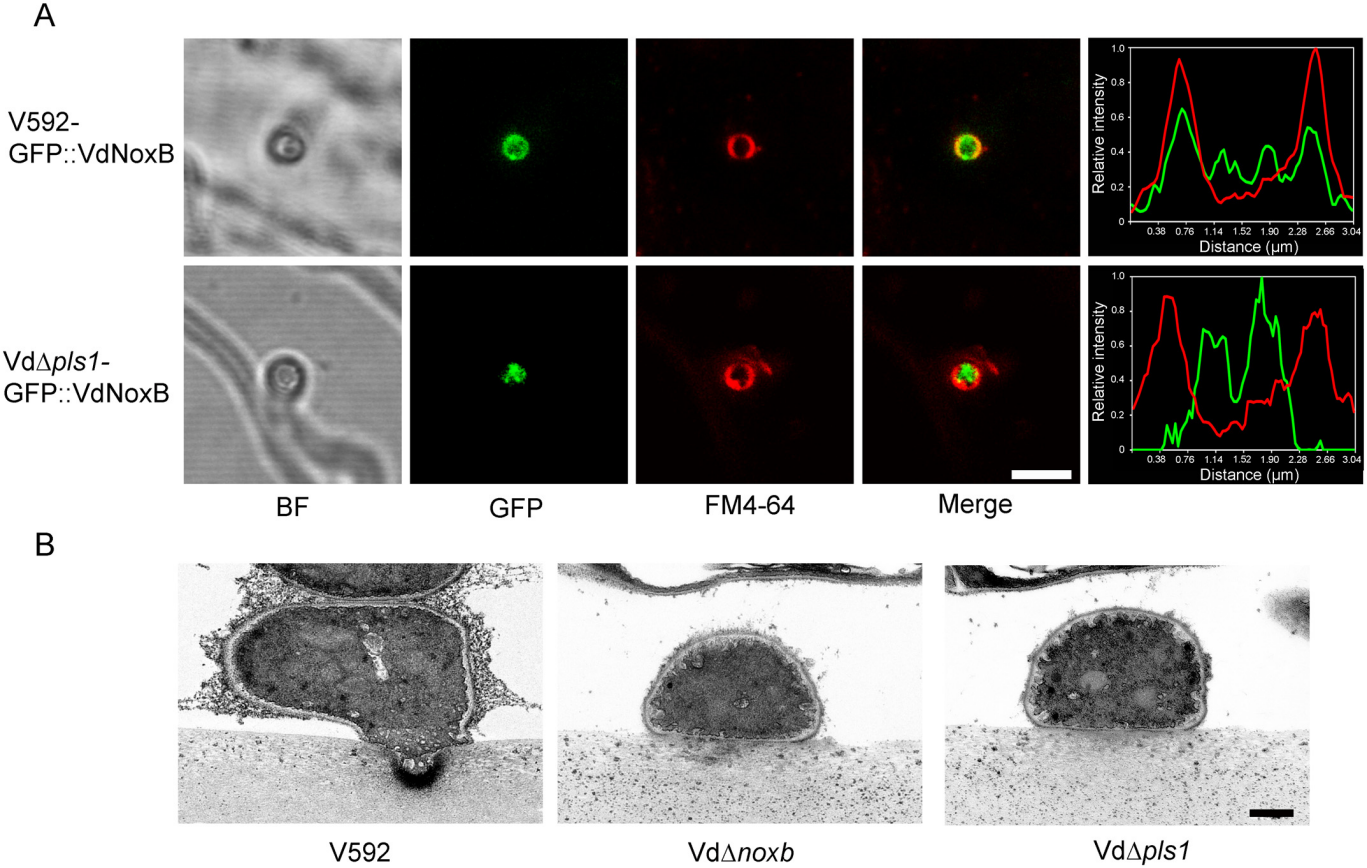
VdPls1-dependent localization and activity of VdNoxB on the membrane of penetration peg. **A.** Micrographs and linescan graph showing GFP::VdNoxB expression in V592 overlapping with the membrane of penetration peg. Mutation of VdPls1 impaired the membrane localization of GFP::VdNoxB. Bar = 5 μm. **B.** Ultrastructural analysis of ROS generation in the hyphopodium of *V*. *dahliae*. V592, Vd*Δnoxb* and Vd*Δpls1* were inoculated on MM medium overlaid with cellophane membrane for 2 days, followed by cerium chloride treatment for TEM observation. Cerium deposits were detected on the membrane at the tip of penetration peg in V592. Deposits were not detected in either mutant strains of Vd*Δnoxb* and Vd*Δpls1*, in which no penetration pegs developed. Bar = 0.5 μm

To determine whether VdPls1 acts as an integral membrane adaptor for the localization and activation of the VdNoxB, we examined the localization of VdNoxB in the *VdPls1* deletion background by introducing the GFP::VdNoxB construct into the Vd*Δpls1* mutant strain. GFP::VdNoxB was also introduced into wild-type V592 as a control. In V592, GFP::VdNoxB was specifically expressed in hyphopodia. When the base of the hyphopodium was observed with CLSM, GFP::VdNoxB was found to localize on and well overlapped with the plasma membrane of the penetration peg (Fig 5A). In Vd*Δpls1* mutant, GFP::VdNoxB was also expressed only in hyphopodia, however, at the base of the hyphopodium, GFP::VdNoxB mainly localized in the cytoplasm without overlapping with the plasma membrane (Fig 5A). In contrast to the tightly GFP::VdNoxB-associated vesicles at the membrane of penetration peg in V592, the GFP::VdNoxB signal in the Vd*Δpls1* mutant was diffuse, which was consistent with the defect in development of penetration peg. This result demonstrates that VdPls1 was required for the plasma membrane localization of VdNoxB and penetration peg formation in the hyphopodium of *V*. *dahliae*.

The involvement of VdPls1 in the regulation of the ROS burst by VdNoxB was then investigated. *V*. *dahliae* hyphae grown on the cellophane membrane were treated with cerium chloride for TEM observation. Fig 5B shows that a large amount of cerium deposits accumulated at the apex of penetration pegs for V592 hyphopodia. In contrast, cerium deposits were not detected in either mutant of Vd*Δnoxb* or Vd*Δpls1* (Fig 5B). Moreover, application of 25μM diphenyleneiodonium (DPI), an inhibitor of flavoenzymes[8], prevented ROS accumulation at the apex of penetration pegs in V592 and cellophane penetration of V592 (S4A and S4B Fig). These results further demonstrate that VdPls1 is required for functional localization of VdNoxB and activate ROS production of the plasma membrane NADPH oxidase on the apex of penetration pegs, revealing that VdPls1 acts as an integral membrane adaptor for the VdNoxB activity in *V*. *dahliae*.

### VdNoxB-dependent ROS production regulates Ca^2+^ signaling in the hyphopodium

**Fig 6:**
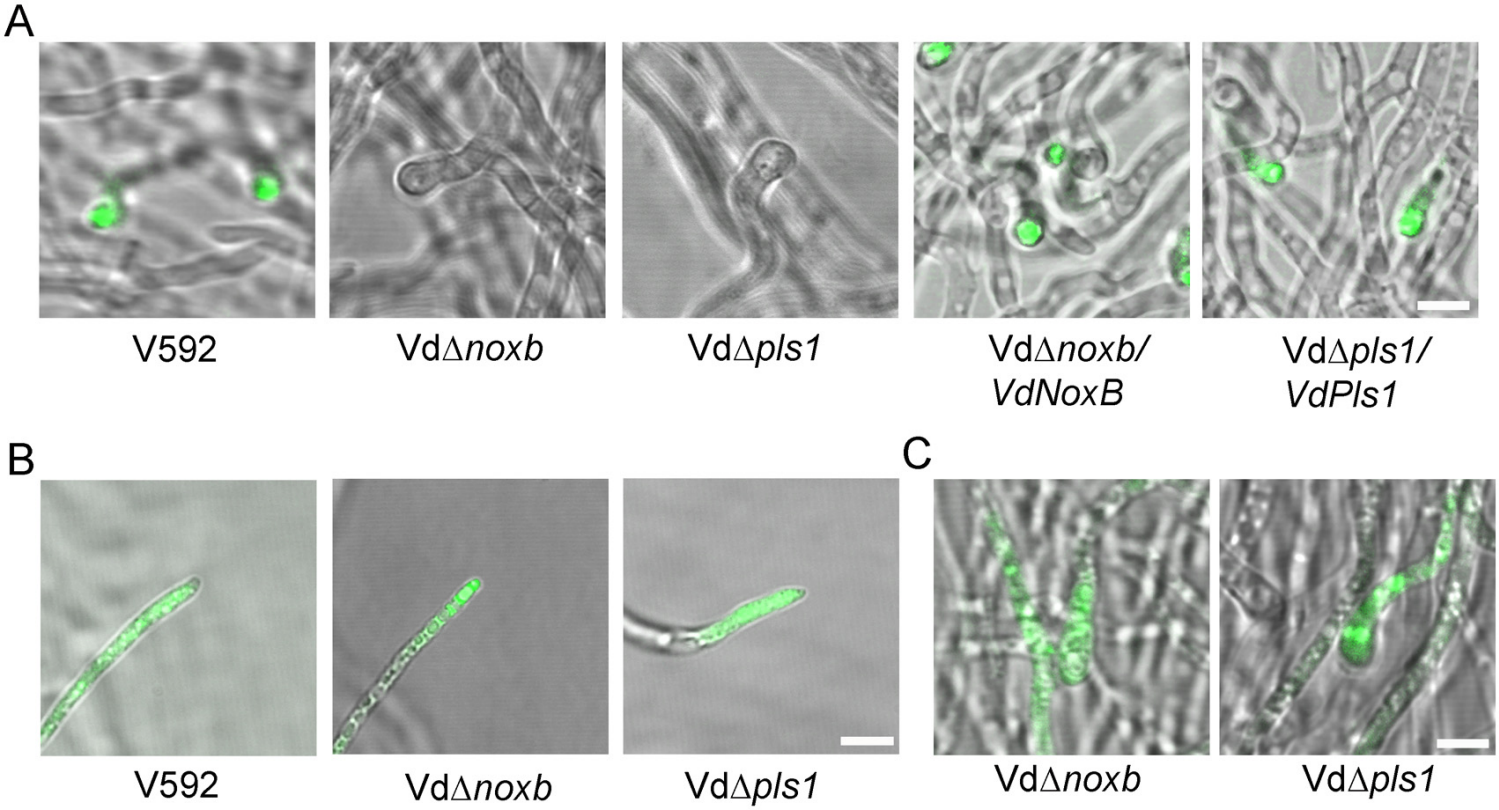
Ca^2+^ detection in hyphopodia and vegetative hyphal tips of wild-type and mutant strains. **A.** Detection of intracellular Ca^2+^ elevation in wild-type and mutant strains. V592, mutant strains Vd*Δnoxb* and Vd*Δpls1*, and complemented strains Vd*Δnoxb*/*VdNoxB* and Vd*Δpls1/VdPls1* were grown on MM medium overlaid with the cellophane membrane for 2 days, and intracellular Ca^2+^ was detected with Fluo-4 AM. A tip-high gradient Ca^2+^ was observed in hyphopodia of V592 and complemented strains but not in the defective hyphopodia of Vd*Δnoxb* or Vd*Δpls1*. Bar = 5μm. **B.** Detection of cytoplasmic Ca^2+^ in vegetative hyphal tips in V592, Vd*Δnoxb* and Vd*Δpls1*. Cytoplasmic Ca^2+^ was detected in wild-type V592 and both mutant strains upon Fluo-4 AM treatment. Bar = 5μm. **C.** Elevation of Ca^2+^ in Vd*Δnoxb* and Vd*Δpls1* upon application of OH·. Vd*Δnoxb* and Vd*Δpls1* grown on cellophane were treated by OH· and cytoplasmic Ca^2+^ was detected with Fluo-4AM. Bar = 5μm.

The ROS burst accompanied vesicles aggregation just behind the membrane of the hyphopodium apex (Fig 5B), reminiscent of the polarized growth of root hairs[31,32,33]. In plants, the apical growth is controlled by ROS-dependent Ca^2+^ influx and gradient concentration formation at the apex[30]. To test whether this mechanism also has specific effects on the establishment of polarized penetration peg formation, we detected the free calcium in the cytoplasm of hyphopodia with Fluo-4AM, an intracellular calcium indicator[38]. As expected, V592 and complemented strains Vd*Δnoxb*/*VdNoxB* and Vd*Δpls1/VdPls1* sets up a tip-high Ca^2+^ gradient in the hyphopodium upon Fluo-4AM treatment (Fig 6A), whereas Vd*Δnoxb* and Vd*Δpls1* fail to accumulate detectable Ca^2+^ in the cytoplasm of the hyphopodium (Fig 6A). However, in wild-type V592 and the mutant strains, Ca^2+^ accumulation was not impaired in vegetative hyphal tips that rapidly grow at the margin of colonies upon Fluo-4AM treatment (Fig 6B). These data indicate the specificity of *VdNoxB* and *VdPls1* in the regulation of the tip-high Ca^2+^ gradient in the hyphopodium, but not in vegetative *V*. *dahliae* hyphae. It has been reported that the superoxide anion produced by a NADPH oxidase can be converted to hydroxyl radicals (OH·) in the presence of transition metals such as Cu^2+^ or Fe^2+^ to mediate Ca^2+^ influx[31]. To confirm the presence of ROS-dependent Ca^2+^ elevation in the hyphopodium, application of OH·, generated by applying a mixture of 2 mM H_2_O_2_, 0.5 mM Cu^2+^ and 0.5 mM ascorbate, elevated the cytoplasmic Ca^2+^ concentration in hyphopodia of both Vd*Δnoxb* and Vd*Δpls1* mutant strains (Fig 6C). This result demonstrates that ROS produced by VdNoxB/VdPls1 were essential for the cytoplasmic free Ca^2+^ elevation in the hyphopodium.

**Fig 7:**
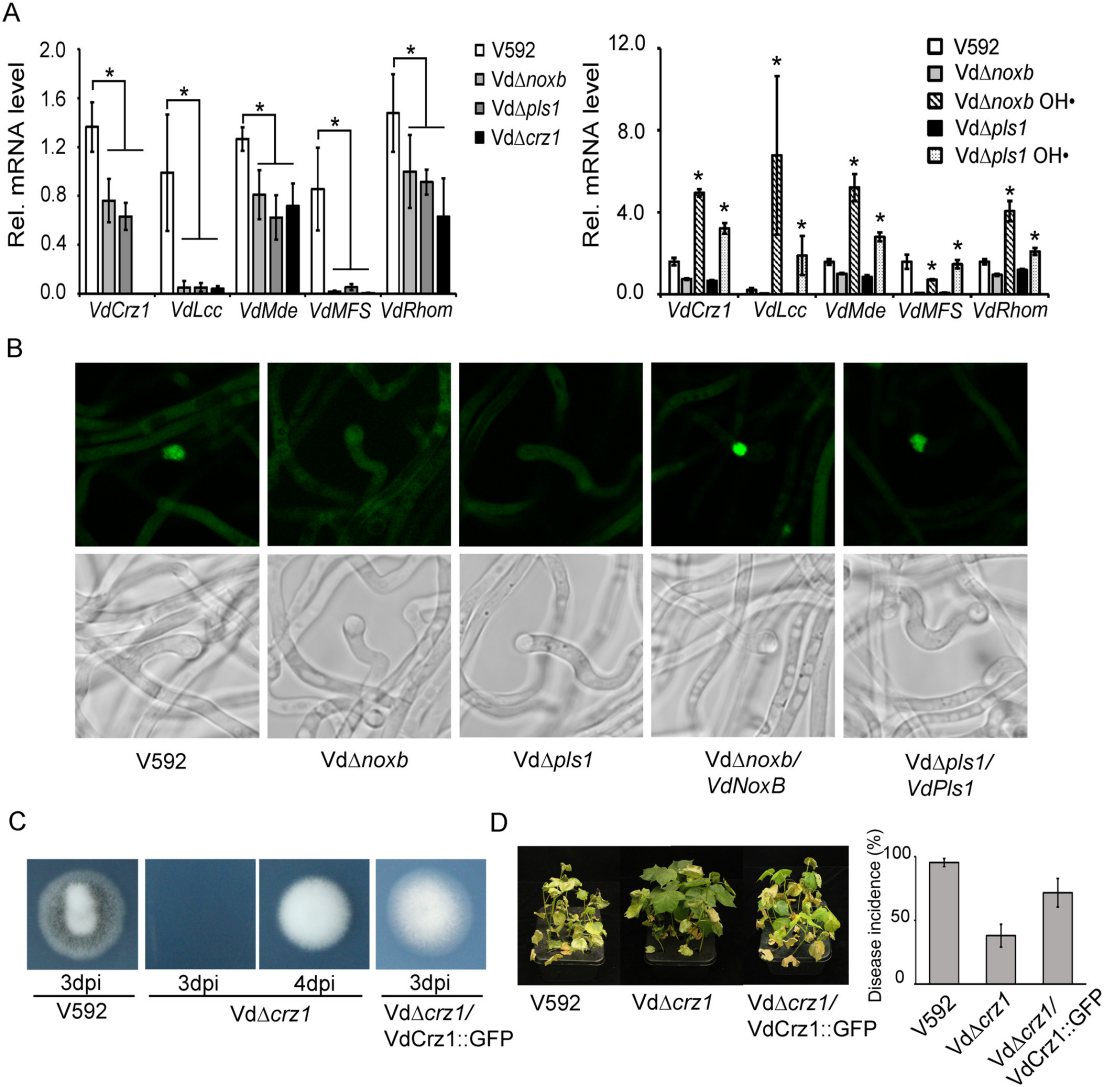
Vd*Crz1* signaling is involved in penetration peg development and pathogenicity of *V*. *dahliae*. **A.** Expression analysis of the Vd*Crz1* signaling-related gene in V592, Vd*Δnoxb*, Vd*Δpls1* and Vd*Δcrz1*(left panel), as well as in Vd*Δnoxb* and Vd*Δpls1* with OH·-treatment (right panel). Total RNA was isolated from hyphae grown on the cellophane membrane for 2 days. Results were obtained from at least three biological repeats. Standard deviations are indicated by the error bars. The asterisks indicate significant differences (P<0.05, one-way ANOVA). **B.** Localization of VdCrz1::GFP in V592, Vd*Δnoxb* and Vd*Δpls1*, and Vd*Δnoxb*/*VdNoxB* and Vd*Δpls1*/*VdPls1* which were grown on MM medium overlaid with the cellophane membrane for 2 days. **C.** Delayed penetration of Vd*Δcrz1* mutant hyphae through the cellophane membrane. Penetration of V592 occurred at 3 dpi, whereas penetration of Vd*Δcrz1* mutant hyphae occurred at 4 dpi. Penetration of Vd*Δcrz1/*VdCrz1::GFP complemented strain restores at 3 dpi. **D.** Delayed development and decreased virulence in Vd*Δcrz1*-infected cotton plants and restored pathogenicity in Vd*Δcrz1/*Crz1::GFP-infected cotton plants.

Calcium ions, as the ubiquitous second messengers, activate many signaling cascades, such as calcineurin-Crz1 signaling, which is the most prominent and best investigated[39,40]. Many orthologues of transcription factor Crz1 have been identified and found to be involved in the regulation of virulence in plant pathogens, including *V*.*dahliae*[41,42,43]. Therefore, we examined whether VdNoxB/VdPls1-dependent Ca^2+^ elevation affected VdCrz1 signaling in *V*. *dahliae*. Wild-type V592 and the Vd*Δnoxb* and Vd*Δpls1* mutant strains were grown on cellophane membrane for hyphopodium induction. Hyphae were collected, and the expression of *VdCrz1* mRNA and its potential targets, including *VdLcc*, *VdMde*, *VdMFS* and *VdRhom*, whose orthologues have been identified in *M*. *oryzae*[44], were examined and compared between the wild-type and mutant strains. Fig 7A showed that *VdCrz1* and its target mRNAs were at lower levels in Vd*Δnoxb* and Vd*Δpls1* compared to that in V592. Application of OH· restored or elevated the *VdCrz1* and its target mRNAs in both Vd*Δnoxb* and Vd*Δpls1* mutant strains (Fig 7A). These data suggest that VdNoxB/VdPls1-dependent Ca^2+^ elevation affected VdCrz1 signaling in *V*. *dahliae* and activated VdCrz1 signaling upon penetration peg induction in wild-type *V*. *dahliae*. Ca^2+^-dependent Crz1 activation is achieved by dephosphorylation and translocation from the cytoplasm to the nucleus of Crz1 by the calmodulin/calcineurin pathway[40]. To examine the migration of VdCrz1 to the nucleus upon hyphopodium induction, GFP was fused at the C-terminus of VdCrz1 to produce VdCrz1::GFP under the oliC promoter and introduced into wild-type V592, the mutant strains Vd*Δnoxb* and Vd*Δpls1* and the complemented strains Vd*Δnoxb*/*VdNoxB* and Vd*Δpls1/VdPls1*. All strains were grown on cellophane membrane for hyphopodium induction. Nuclear targeting of VdCrz1::GFP was observed in hyphopodia for V592 and Vd*Δnoxb*/*VdNoxB* and Vd*Δpls1/VdPls1*, but not for Vd*Δnoxb* and Vd*Δpls1* mutant strains (Fig 7B), demonstrating the VdNoxB/VdPls1-dependent ROS production coupled with Ca^2+^ elevation have a specific effect on VdCrz1 activation in hyphopodium. We then generated a knockout mutant for *VdCrz1* for penetration peg formation and pathogenicity assays. Similar to the Vd*Δnoxb* and Vd*Δpls1* knockout mutants, Vd*Δcrz1* mutant colonies exhibited higher hyphal growth rate and the loss of melanin production on PDA plates (S5 Fig). The Vd*Δcrz1* mutant displayed delayed penetration peg formation and greatly reduced virulence in cotton plants compared to wild-type V592 (Fig 7C and 7D). Delayed but not inhibited penetration of the cellophane membrane by the Vd*Δcrz1* mutant suggests the existence of other signaling cascades that are also activated by ROS-Ca^2+^ elevation in hyphopodium for penetration peg development. Both penetration ability and pathogenicity for complemented strain Vd*Δcrz1/*VdCrz1::GFP were restored (Fig 7C and 7D). Taken together, our results demonstrate that VdNoxB/VdPls1-mediated ROS production activates VdCrz1 signaling through Ca^2+^ elevation in the hyphopodium to regulate penetration peg formation during the initial colonization of cotton roots.

## Discussion

The tetraspanins are a family of proteins that cross the membrane four times and are abundant in the membranes of various types of endocytic organelles and in exosomes-different sizes of vesicles- that are released by many cells[45]. In mammals, it has been reported that tetraspanins act as molecular facilitators to modulate the activities of their associated molecules[45]. The first and only evidence for the essential role of the tetraspanin TSP-15 as a key component in ROS generation is from a study on the induction of H_2_O_2_ by DUOXs, a member of the Nox family, in *Caenorhabditis elegans*[46]. However, the role of the fungal tetraspanin Pls1 as an integral membrane adaptor protein for the catalytic activity of the Nox has not been conclusively shown[13].

In the present study, we provided several lines of evidence demonstrating that the tetraspanin VdPls1 functions as an integral membrane adaptor protein for the activity of the NoxB, for the generation of ROS specifically in the hyphopodium to mediate the tip-focused cytoplasmic Ca^2+^elevation that is important for penetration peg development in *V*. *dahliae*. (1) Mutants knocked out for either *VdNoxB* or *VdPls1* lose virulence due to the defect in the initial colonization in cotton plants, resulting from the inability to form penetration pegs. (2) Our results showed the identical expression pattern of *VdNoxB* and *VdPls1*, and the co-localization of VdNoxB and VdPls1 in hyphopodium at the penetration peg emerging site. (3) Knocking out *VdPls1* resulted in defective plasma membrane localization of VdNoxB and ROS production. (4) Both VdPls1 and VdNoxB were required for the ROS-mediated tip-high Ca^2+^ gradient and activation of Ca^2+^ signaling specifically in the hyphopodium to regulate penetration peg formation in *V*. *dahliae*.

### *V*. *dahliae* differentiates the hyphopodium as an infection structure to develop penetration peg that is regulated by VdNoxB and VdPls1

Unlike the conspicuous fungal appressoria, which differentiate rapidly after conidia germination when they are inoculated on plant leaves, *V*. *dahliae* conidia always germinate germ tubes on the root surface, followed by extension of hyphae[4,7]. Large numbers of hyphae grow and wrap the root surface. Only a few hyphae tightly adhering on the root surface are able to successfully invade the root cortex by penetrating intercellularly into the epidermal cells and further advancing the internal infection[4,7]. This is consistent with the finding that only a few of *V*. *dahliae* hyphae were induced to differentiate the hyphopodia for polarized growth of the penetration pegs on the cellophane membrane.

Although ROS-mediated polarity has been widely studied in animals[47] and plants[48], its roles in the polarity of filamentous fungi has only recently emerged[49]. The *Pls1* homologues of several fungal pathogens, for example, *M*. *grisea*, *B*. *cinerea* and *Colletotrichum lindemuthianum*, were found to be expressed during appressoria development and are required for appressorium-mediated penetration of host plant leaves[50,51,52]. The Pls1 protein was found to localize to the plasma membrane and in vacuoles in appressoria and associated with the ER[29,50]. During root colonization by *M*. *oryzae*, the *Pls1* also expressed in hyphopodium[6], implying that Pls1-dependent signaling complexes are active during both leaf and root colonization. Overlapping phenotypes of Pls1 and NoxB deletion mutants of *P*. *anserina* and *B*. *cinerea* suggested Pls1 to be a NoxB-interacting partner[28,29]. In *M*. *grisea*, the *Nox1* (*NoxA*) and *Nox2* (*NoxB*) genes were identified as sources of ROS production[8], and the function of Nox2 is also necessary for initiation of polarized growth of the penetration peg at the appressorium base[9,10]. In this study, both GFP::VdNoxB and GFP::VdPls1 were readily observed in hyphopodia. Knocking out either *VdNoxB* or *VdPls1* did not affect the formation of hyphopodia, despite being incapable of development of penetration pegs, indicating the neither these gene is required for the development of hyphopodia, but both are essential for penetration peg formation. Our data provide for the first time the molecular feature for accurate identification of hyphopodium, an appressorium-like infectious structure, in *V*. *dahliae*.

### VdPls1 activates VdNoxB at the base membrane of hyphopodia to regulate penetration peg formation

ROS production by Nox is under strict spatial regulation within the cell[53]. In human polymorphonuclear neutrophils, production of O^2–^ is dependent on translocation of the oxidase subunits, including the membrane component gp91^*phox*^/p22^*phox*^ and the cytosolic components p47^*phox*^-p67^*phox*^-p40^*phox*^ and Rac2 from the cytosol or specific granules to the plasma membrane. In resting status, however, 85% of gp91^*phox*^/p22^*phox*^ complexes remain in the membrane of the specific granules and secretory vesicles[54,55,56,57]. Similarly, we found that small vesicles in the hyphopodium contain the majority of VdNoxB and VdPls1. Only when both VdNoxB and VdPls1 highly co-localized with the plasma membrane at the penetration peg emergence site was oxidase activity possible. Due to a small number of hyphopodia were induced and possible dynamic effect, it was hard to provide substantive evidence for the interaction of VdNoxB and VdPls1. However, high co-localization of both VdNoxB and VdPls1 with the plasma membrane at the penetration peg where the clear florescent signal of BiFC assay was also observed implied interaction between two proteins probably only at the base of hyphopodium. We also found that, in the Vd*Δpls1* mutant strain, VdNoxB-associated vesicles aggregated to the base of the hyphopodium were incapable of targeting to plasma membrane. Our data reveal that the *V*. *dahliae* tetraspanin Pls1 protein functions as an integral membrane adaptor protein for the recruitment of the NoxB to plasma membrane. It has been suggested that animal tetraspanins could be associated with other membrane proteins in complexes that might include integrins[45]. Our data demonstrate that such membrane complexes are present in *V*. *dahliae*, which is composed of the catalytic subunit VdNoxB and the adaptor protein VdPls1, and is essential for the catalytic activity of the NADPH oxidase and responsible for the ROS burst and penetration peg differentiation. Together with the previous finding in *Epichloë festucae*[17] that cytosolic components of fungal Nox interact at hyphal tips and are essential for Nox activity, we proposed that Nox activity in fungi is also under strict regulation, including the translocation of membrane and cytosolic subunits to target sites, such as penetration peg membranes, which implies that a conservative regulation mechanism of the Nox complex exists in eukaryotes.

### ROS-dependent generation of tip-focused cytoplasmic Ca^2+^ accumulation is important for penetration peg development

Formation of a penetration peg involves a switch from isotropic growth (swelling) to apical hyphal growth which is essential for the redirection of growth toward the host cell. The indispensability of *NoxB* for this repolarization process is consistent with the role of ROS in the establishment of polarity[49]. In this study, we found that VdPls1, in conjunction with VdNoxB, establishes a specialized membrane microdomain that facilitates the generation of ROS to regulate polarized penetration peg formation by generating a tip-focused Ca^2+^ gradient in the hyphopodium. Similarly, ROS-Ca^2+^ the mechanism has been defined in the regulation of the highly polarized growth of root hairs and pollen tubes[31,58], where the tip-focused Ca^2+^ gradient is involved in modulating F-actin dynamics[59] and stimulating exocytosis[60,61] to sustain tip growth. Consistent with this, the recent studies in *M*. *oryzae* also showed that *Nox2* (*NoxB*) in penetration peg formation is based on remodeling of the F-actin cytoskeleton[9] and exocytosis apparatus[10]. We found that Vd*Δnoxb* and Vd*Δpls1* mutants do not affect Ca^2+^ accumulation in vegetative hyphal tips. It has also been reported that no detectable difference exists in superoxide production between the vegetative hyphal tip of WT and the Δnox2 mutant in *M*. *oryzae*[8]. These findings indicate that the mechanisms for creating ROS vary between vegetative hyphae and invasive hyphae and highlight the specific role of *NoxB* in Ca^2+^ regulation during hyphopodium development. In addition, we found that conservative Ca^2+^-Crz1 signaling is involved in penetration peg formation, suggesting that Ca^2+^ as a second messenger, transduces the apical ROS signal to regulate morphogenesis of penetration pegs. Therefore, we suggest that ROS-dependent Ca^2+^ accumulation is a hallmark and common regulatory mechanism for apical growth in eukaryotes.

**Fig 8:**
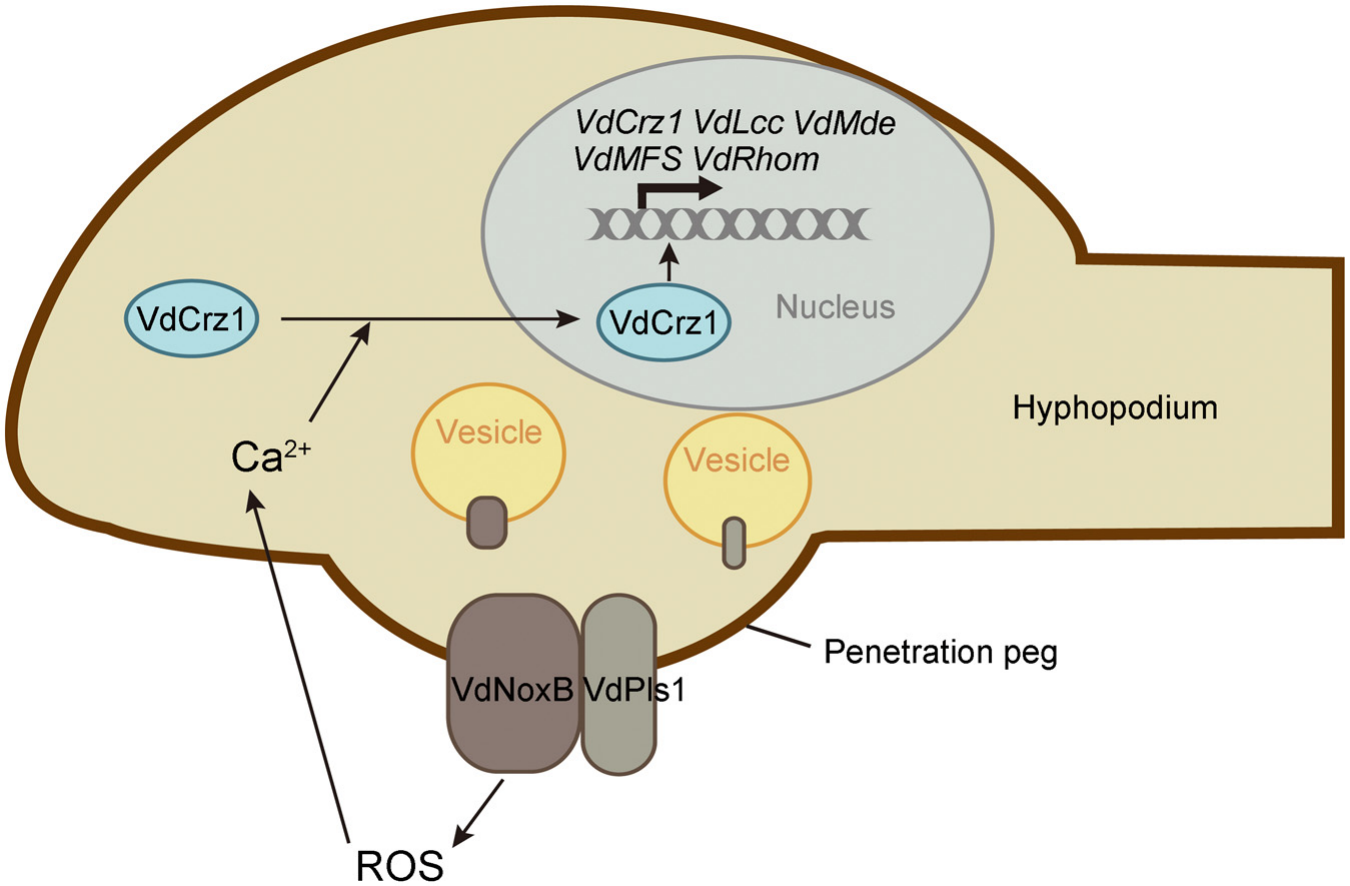
Schematic overview of *VdNoxB/VdPls1*-depedent ROS-Ca^2+^ signaling in *V*. *dahliae*. Hyphopodia are infection structures of *V*. *dahliae*. ROS produced by VdNoxB/VdPls complex elevate cytoplasmic Ca^2+^ in hyphopodia. Accumulation of Ca^2+^ activates nuclear targeting of VdCrz1 transcription factor and triggers VdCrz1 signaling to regulate penetration peg development.

In summary, we provide, for the first time, the molecular features of the infectious structure, the hyphopodium, in *V*. *dahliae*. As shown the simple schematic in Fig 8, our data demonstrate that the tetraspanin VdPls1 functions as an integral membrane adaptor protein for the assembly of the VdNoxB to plasma membrane for local ROS production, and ROS-Ca^2+^ signaling in the hyphopodium plays key roles in regulating polarized cell expansion-penetration peg formation and pathogenicity in *V*. *dahliae*. These findings imply that targeting ROS-Ca^2+^ signaling is likely to be an effective means of controlling the devastating Verticillium wilt disease at its initial stages.

## Materials and Methods

### Fungal isolates, culture conditions and nucleic acid analysis

*Verticillium dahliae* V592, a virulent defoliating isolate, was used as a host strain and a wild-type control. The other transformants used in this study are listed in S1 Table. Cultures were reactivated on potato dextrose agar (PDA) medium. Conidia production for infection assays were cultured in liquid Czapek-Dox medium. Minimal medium (glucose at 2g/liter, NaNO_3_ at 2 g/liter, KH_2_PO_4_ at 1g/liter, MgSO_4_-7H_2_O at 0.5 g/liter, KCl at 0.5 g/liter, citrate at 10 mg/liter, ZnSO_4_-7H_2_O at 10 mg/liter, FeSO_4_-7H_2_O at 10 mg/liter, NH_4_Fe(SO_4_)_2_-12H_2_O at 2.6 mg/liter, CuSO_4_-5H_2_O at 0.5 mg/liter, MnSO_4_-H_2_O at 0.1 mg/liter, H_3_BO_3_ at 0.1 mg/liter, Na_2_MoO_4_-2H_2_O at 0.1 mg/liter and agar at 20 g/liter) was used for penetration assays. Incubation took place at 26°C.

For nucleic acid extraction and blotting, Fungal isolates were grown in the liquid Czapek-Dox medium for 2 days with shaking at 200 rpm, 26°C, dark condition, and the resulting mycelium was harvested by centrifugation at 12000 rpm for 1 min. DNA and RNA isolation and hybridization was described previously [36]. PCR, gel electrophoresis, and DNA sequencing were performed as described by Sambrooket al[62]. For Southern blot analysis, 20 μg of genomic DNA was completely digested with proper restriction enzymes, then separated by electrophoresis on an agarose gel and transferred onto a nylon membrane. Gene specific probes were amplified with primers listed in S2 Table and labeled with ^32^P using the Random Prime Labeling System Rediprime II (GE Healthcare, Piscataway, NJ, USA). DNA gel blots were performed as described by Sambrook et al. [62]. 40 μg total RNA was used for Northern blot analysis. Hybridization was carried out as described by Sambrook et al. [62] with the same probes used in Southern blot.

### Penetration assays, fungal recovery and pathogenicity assays

For penetration assays, sterilized cellophane membrane (DINGGUO, Beijing, China) was overlaid onto MM medium. The cultures were incubated on the cellophane membrane for various lengths of time. The membranes were removed, and hyphae were observed in the underlying medium to determine if there were any breaches of the cellophane. For DPI treatment, fungi were grown on MM medium overlaid with cellophane membrane for 1 day. Then, the membrane was transferred to MM medium with 25 μM DPI (Sigma) followed by another 2 days incubation and removed for penetration detection. The experiments were repeated independently at least three times.

The fungus was recovered from infected cotton as follows: the stem sections above cotyledons of cotton plants were taken at 30 days after inoculation and surface-sterilized for 1 min in 70% ethanol, followed by 60 min in 10% hydrogen peroxide. The samples were then rinsed three times with sterile water, cut into 1 cm slices and cultured at 26°C on PDA medium.

Upland cotton was inoculated with V592 and transformants for the infection assays, using our laboratory unimpaired root-dip inoculation method [36]. Disease severity was counted by the percentage of cottons that showed wilting symptom at 30 dpi after inoculation. The infection assays for each mutant colony were repeated at least three times.

### Construction and transformation

To generate the knockout plasmids pKOVdNoxB, pKOVdPls1 and pKOVdCrz1, upstream and downstream genomic sequences were amplified with the following primer pairs: KONoxBup-s/a, KONoxBdn-s/a, KOPls1up-s/a, KOPls1dn-s/a, KOCrz1up-s/a and KOCrz1dn-s/a (S2 Table). Both sequences were inserted into a position flanking of the hygromycin resistant cassette of the vector pGKO with the USER enzyme to generate knock-out plasmids, and transformation was performed as described previously[35]. To generate the complementary plasmids of pNEO-NoxBcom and pNEO-Pls1com, wild-type *VdNoxB* and *VdPls1* genes including native promoter and terminator were amplified from V592 genome with primer pairs of NoxBup-s/NoxBdn-a and Pls1up-s/Pls1com-a (S2 Table). Both fragments were inserted into HindIII/EcoRI-linearized pNEO binary vector. The pNEO binary vector was created by insertion of G418 resistant cassette amplified with Neo-s/a primers from pKOV21 into XbaI/KpnI-digested pSULPH-GFP[36]. pNEO-NoxBcom and pNEO-Pls1com plasmids were transformed into knockout mutants of Vd*Δnoxb* and Vd*Δpls1*, respectively, to produce complemented strains, Vd*Δnoxb* ***/*** *VdNoxB* and Vd*Δpls1/VdPls1*. Transformants were selected on PDA medium with G418 at 40 mg/liter. V592, Vd*Δnoxb* and Vd*Δpls1* mutant strains, as well as Vd*Δnoxb*/*VdNoxB* and Vd*Δpls1*/*VdPls1* complemented strains were transformed with pSULPH-GFP to produce corresponding strains expressing GFP. Transformants were selected on PDA medium with chlorimuron-ethyl at 100 mg/liter.

To obtain GFP-VdNoxB and GFP-VdPls1 fusion plasmids, GFP and *VdNoxB* and *VdPls1* promoter sequences, and downstream sequences including coding sequence and native terminator of *VdNoxB* and *VdPls1* were amplified with the primers listed in S2 Table. The corresponding fragments with homologous recombination sequences were ligated into HindIII/EcoRI-linearized pNEO and pSULPH-GFP binary vectors, respectively, based on homologous recombination cloning (ClonExpressMultiS One Step Cloning Kit, Vazyme, China). The resulting GFP::VdNoxB and GFP::VdPls1 plasmids were transformed into Vd*Δnoxb* and Vd*Δpls1*, respectively. GFP::VdNoxB was also transformed into V592 and Vd*Δpls1*. Transformants were selected on PDA medium with G418 or chlorimuron-ethyl.

For Y2H assay, the full length ORF of *VdNoxB*, *VdPls1* and *VdAR* were amplified from cDNA of V592 with the primers listed in S2 Table and inserted into Sfi I-linearized pPR3-N or pBT3-N Y2H vector. *VdAR* is the homologue of yeast membrane protein, adiponectin receptor Izh2, and was used as the negative control.

To generate VC::VdNoxB and VN::VdPls1 for BiFC assays, N- and C-terminal fragments of Venus were amplified with primers of VN-s/a and VC-s/a. The PacI-SpeI fragments of VC and VN were ligated into PacI/SpeI digested GFP-VdNoxB and GFP-VdPls1, respectively, to replace the GFP fragment. To create VdMsb2::GFP construct, VdMsb2 genomic sequence, GFP, oliC promoter and gluc terminator were amplified with the primers listed in S2 Table and ligated into HindIII/EcoRI-linearized pNEO vector based on homologous recombination cloning (ClonExpressMultiS One Step Cloning Kit, Vazyme, China). pNAH-Grx1-roGFP2 plasmid was used as the template for oliC promoter and gluc terminator amplification. For the VdMsb2::VC plasmid, the GFP fragment of VdMsb2::GFP was replaced with C-terminal fragments of Venus digested by PacI/SpeI. VC::VdNoxB/VN::VdPls1 or VdMsb2::VC/VN::VdPls1 was co-transformed into V592 and transformants with G418 and chlorimuron-ethyl resistance were selected.

To generate 3Flag::VdPls1 construct for co-IP assay, 3Flag fragment was cloned with primers of 3Flag-s/a, followed by ligation into PacI/SpeI-digested GFP-VdPls1 to replace GFP fragment. 3Flag::VdPls1 was transformed into V592-GFP::VdNoxB strain and transformant was selected with Chlorimuron-ethyl.

To obtain VdCrz1::GFP fusion plasmid, VdCrz1 genomic sequence, GFP, oliC promoter and gluc terminator were amplified with the primers listed in S2 Table and ligated into HindIII/EcoRI-linearized pSULPH-GFP vector based on homologous recombination cloning (ClonExpressMultiS One Step Cloning Kit, Vazyme, China). The resulting plasmid was transformed into Vd*Δcrz1*to get the complemented strain Vd*Δcrz1/VdCrz1*. VdCrz1::GFP was also introduced into wild-type V592, the mutant strains Vd*Δnoxb* and Vd*Δpls1*, and the complemented strains Vd*Δnoxb*/*VdNoxB* and Vd*Δpls1/VdPls1*.

### Interaction assays

For Y2H assay, VdNoxB and VdPls1 were tested with a split ubiquitin based membrane yeast two-hybrid system. Constructs of pPR3N-NoxB, pBT3N-Pls1 and respective control vector were co-transformed in Yeast NMY51 and cultured on the SD-Leu-Trp-His-Ade medium adding 10 mM 3-AT and 80 mg/liter X-Gal for 3–4 days at 30°C. pBT3N-Pls1/pOST1-NubI was used as positive control. pBT3N-Pls1/pPR3-N and pPR3N-NoxB/pBT3N-AR were used as negative control.

For BiFC assay, VC::VdNoxB/ VN::VdPls1 andVdMsb2::VC/VN::VdPls1 were transformed into V592. Transformants were inoculated on MM medium overlaid with cellophane for 2 days to induce hyphopodium development. Then, the cellophane with fungal colonies was removed for CLSM observation.

For co-IP assay, V592 expressing GFP::VdNoxB and 3Flag::VdPls1 was grown on MM medium overlaid with cellophane for 2 days. Mycelium was harvested and protein was extracted by lysis buffer (20 mM Tris-HCL pH 8, 150 mM NaCl, 1% Triton X-100 and 1 x Protease inhibitor (Roche)). Whole protein extract was incubated with GFP-Trap beads (ChromoTek) or Flag M2 beads (Sigma) overnight at 4°C. Detection of proteins was done with Flag antibody (1:1000 diluted, Sigma) or GFP antibody (1:1000 diluted, Santa).

### Confocal laser scanning microscopy and transmission electron microscopy

To observe the infection process of *V*. *dahliae*, hydroponic cotton roots inoculated for 3 or 5 days were sectioned by hand and plant cell walls were counterstained with propidium iodide. The mycelium grown on cellophane for 2 days and was used for hyphopodium detection and protein localization assays. The plasma membrane and the ER were stained with FM4-64 (ThermoFisher) and ER-Tracker Blue-White DPX (ThermoFisher) according to the manufacturer's protocol. Fluorescent photographs were taken using a Leica SP8 confocal laser scanning microscope system under 63× or 100×oil immersion objective lenses. During microscopy, 488-nm and 514nm laser were used to excite GFP and Venus, respectively. All images were captured with a Leica hybrid detector and analyzed with Leica LAS AF software.

For TEM observation, hyphopodium formation on the cotton root was detected first by CLSM at 3 dpi, then the root with hyphopodium was cut into slices less than 1 mm thick. These slices were fixed immediately in 2.5% glutaraldehyde, buffered with PBS (pH 7.4) at 4°C overnight, washed with the same buffer four times and post-fixed with 1% osmium tetroxide for 1 h. Then, the dehydration was performed in an acetone series (50%, 75%, 85%, 95%, 100%), and the slices were embedded in Spurr’s resin mixture. Ultrathin serial sections (70 nm thickness) were cut from resin blocks, followed by uranyl acetate staining, and observed with JEM-1400 electron microscope.

### ROS and Ca^2+^ detection

For ROS detection with TEM, cellophane with mycelium at 2dpi was stainedby5 mM cerium chloride solution buffered with 50 mM 3-(*N-*morpholino) propanesulphonic acid (MOPS, pH 7.4) at room temperature for 1 h, followed by preparation for TEM observation as descripted above. Samples treated with 25 μM DPI (Sigma) for 2 hours before cerium staining were used as negative controls.

For cytoplasmic Ca^2+^ detection, a stock solution of Fluo-4AM (ThermoFisher), 4 mM in dimethyl sulfoxide (DMSO), was prepared and diluted with liquid MM medium to make a 5 μM work solution. Mycelium grown on cellophane for 2 days was loaded with 5 μM Fluo-4AM for 30min at room temperature, washed three times and observed by CLSM, according to the manufacturer’s instructions. To evaluate the role of ROS in cytoplasmic Ca^2+^ accumulation, OH· were generated by addition a mixture of 2mM H_2_O_2_, 0.5 mM Cu^2+^ and 0.5 mM ascorbate and applied to mycelium for 30min following Fluo-4AM loading.

### Quantitative real-time-PCR

Total RNA was isolated from mycelium grown on cellophane for 2 days. For OH· treatment, the mycelium was applied with a mixture of 2mM H_2_O_2_, 0.5 mM Cu^2+^ and 0.5 mM ascorbate for 30 min and collected for RNA isolation. The residual DNA was removed from the total RNA using TURBO DNase (ThermoFisher). cDNA was reverse transcribed using HiScript II Q RT Supermix (Vazyme) and qRT-PCR was performed using ChamQ SYBR qPCR Master Mix (Vazyme) in Bio-Rad CFX96 Real-Time system. Transcription levels of the target genes were quantified relative to the constitutively expressed elongation factor 1-α of *Verticillium dahliae* (*VdElf*). Gene-specific primers are listed in S2 Table. Biological replicates were performed at least three times.

### Accession numbers

GenBank accession numbers for genes in this study: *VdNoxB* (VDAG_09930), *VdPls1* (VDAG_01769), *VdMsb2* (KM032761), *VdAR*(VDAG_03172), *VdCrz1* (VDAG_03208), *VdLcc* (VDAG_00189), *VdMde* (VDAG_07056), *VdMFS* (VDAG_05637), *VdRhom* (VDAG_08343), *VdElf*(VDAG_02559), *MoPls1* (MGG_12594), *MoNox2* (MGG_06559).

## Supporting Information

S1 Fig Construction of knockout mutants Vd*Δnoxb* and Vd*Δpls1*. **A.** Schematic representation of the homologous recombination event involved in the targeted replacement of Vd*NoxB* and Vd*Pls1*. **B.** PCR identification of the knockout mutant with the primers IDE-s and IDE-a indicated in A. **C.** Southern blot analysis of targeted gene deletion mutants. Bgl II digested genomic DNA from V592 wild type strain and two putative Δ*noxb* transformants were gel fractionated (left) and blotted (right) with the probe indicated in the schematic diagram. Sal I digested genomic DNA from V592 wild type strain and two putative Δ*pls1* transformants were analyzed as described above. **D.** Northern blot analysis of the expression of Vd*NoxB* and Vd*Pls1* in knockout mutants with the probe used for southern blot analysis.
(TIF)

S2 Fig Phenotype analysis of Vd*Δnoxb*/GFP::VdNoxB and Vd*Δpls1*/GFP::VdPls1. **A.** Penetration assay of complemented strains of Vd*Δnoxb*/GFP::VdNoxB and Vd*Δpls1*/VdGFP::Pls1. ‘Above’ and ‘Below’ show colonies grown on cellophane membrane and medium after the membrane was removed, respectively. **B.** Observation of penetration peg development on the cellophane membrane at 2 dpi. Penetration pegs (the dark pin) were observed from the hyphopodia, the second row was focused at 5 μm below the first row. Bar = 5 μm. **C**. Restoration of pathogenicity of Vd*Δnoxb*/GFP::VdNoxB and Vd*Δpls1*/GFP::VdPls1 on cotton plants. Photographs were taken at 30 dpi. **D.** Schematic representation of the GFP::VdNoxB and GFP::VdPls1 constructs. PCR confirmation of transformants with the primers indicated in schematic diagram.
(TIF)

S3 Fig Possible interaction of VdNoxB and VdPls1 in hyphopodia. **A.** Yeast two-hybrid assay showing direct interaction of VdNoxB and VdPls1. Constructs of pPR3N-NoxB, pBT3N-Pls1 and respective control vector were co-transformed in Yeast NMY51 and cultured on the SD-Leu-Trp-His-Ade/10 mM 3AT/X-Gal medium with 1, 1:10, 1:100, and 1:1000 dilutions. Positive control: pBT3N-Pls1/pOST1-NubI. Negative control: pBT3N-Pls1/pPR3-N and pPR3N-NoxB/pBT3N-AR. B. BiFC assay showing interaction of VdNoxB and VdPls1 in vivo. VN-VdNoxB and VC-VdPls1 were transformed in V592 and Venus signal on the membrane of penetration peg was detected. Plasma membrane was stained with FM4-64. VdMsb2::VC was co-transformed with VN::VdPls1 as the negative control. Bar = 5μm. **C**. Localization of VdMsb2::GFP. Localization of VdMsb2 in V592-Msb2::GFP strain incubated on cellophane membrane. The image was taken at 2 dpi and shows that VdMsb2 localized on the membrane of fungal hyphae and aggregated with the membrane of penetration peg (indicated by an arrow). Bar = 5μm. **D**. Immunoblot of the failed co-immunoprecipitation result. V592 expressing GFP::VdNoxB and 3Flag::VdPls1 was grown on MM medium overlaid with cellophane for 2 days to conduct Co-IP assay. **E.** Schematic representation of the BiFC assay related constructs. PCR confirmation of positive transformants with the primers indicated in schematic diagram.
(TIF)

S4 Fig ROS detection at the penetration peg with DPI treatment. **A.** Detection of cerium deposits by TEM. V592 was treated with DPI 2 hours before cerium chloride staining, and the control was treated with DMSO. Cerium deposits were detected on the membrane at the tip of penetration peg in V592 treated with DMSO. DPI prevented ROS accumulation at the apex of penetration peg. Bar = 0.5 μm. **B.** V592 penetration assay with DPI treatment. V592 was grown on cellophane for 1 day to develop hyphopodia followed by transfer of cellophane to MM medium containing DPI for another 2 days. MM medium containing DMSO was used as a control. The image shows growth of a V592 colony on MM medium with a cellophane layer (above) and removal of the cellophane membrane (below). Photographs in the first row were taken at 7 dpi. V592 penetration of cellophane was blocked on MM medium containing DPI.
(TIF)

S5 Fig Identification of knockout mutant and colony morphology of Vd*Δcrz1*. **A.** PCR identification of the *VdCrz1* knockout mutant with primers Crz1IDE-s and Crz1IDE-a as indicated in S1A Fig. **B.** Southern blot analysis of *VdCrz1* deletion mutant. Kpn I digested genomic DNA from V592 wild type strain and putative Δ*crz1* transformant were gel fractionated (left) and blotted (right) with the probe indicated in the schematic diagram. **C**. Colony morphology of V592, Vd*Δcrz1* and Vd*Δcrz1/*VdCrz1::GFP on PDA plates after 2 weeks post-incubation. D. Schematic representation of the VdCrz1::GFP construct. PCR confirmation of transformant with the primers indicated in schematic diagram.
(TIF)

S1 Table List of *Verticillium dahliae* strains used in this study. (DOCX)

S2 Table List of primers used in this study. (DOCX)

## References

[1] Pegg GF (1989) Pathogenesis in vascular disease of plants, Pp. 51–94; Tjamos EC, Beckman C, editors: Springer-verlag, Berlin, Germany.

[2] Garber R, Houston B (1966) Penetration and development of Verticillium albo-atrum in the cotton plant. Phytopathology 56: 1121–1126.

[3] Gerik J, Huisman O (1988) Study of field-grown cotton roots infected with Verticillium dahliae using an immunoenzymatic staining technique. Phytopathology 78: 1174–1178.

[4] Zhao P, Zhao YL, Jin Y, Zhang T, Guo HS (2014) Colonization process of Arabidopsis thaliana roots by a green fluorescent protein-tagged isolate of Verticillium dahliae. Protein Cell 5: 94–98. 10.1007/s13238-013-0009-9 24481631 PMC3956967

[5] Sesma A, Osbourn AE (2004) The rice leaf blast pathogen undergoes developmental processes typical of root-infecting fungi. Nature 431: 582–586. 15457264 10.1038/nature02880

[6] Tucker SL, Besi MI, Galhano R, Franceschetti M, Goetz S, et al (2010) Common genetic pathways regulate organ-specific infection-related development in the rice blast fungus. Plant Cell 22: 953–972. 10.1105/tpc.109.066340 20348434 PMC2861474

[7] Reusche M, Truskina J, Thole K, Nagel L, Rindfleisch S, et al (2014) Infections with the vascular pathogens Verticillium longisporum and Verticillium dahliae induce distinct disease symptoms and differentially affect drought stress tolerance of Arabidopsis thaliana. Environmental and Experimental Botany 108: 23–37.

[8] Egan MJ, Wang ZY, Jones MA, Smirnoff N, Talbot NJ (2007) Generation of reactive oxygen species by fungal NADPH oxidases is required for rice blast disease. Proc Natl Acad Sci U S A 104: 11772–11777. 17600089 10.1073/pnas.0700574104 PMC1913907

[9] Ryder LS, Dagdas YF, Mentlak TA, Kershaw MJ, Thornton CR, et al (2013) NADPH oxidases regulate septin-mediated cytoskeletal remodeling during plant infection by the rice blast fungus. Proceedings of the National Academy of Sciences 110: 3179–3184. 10.1073/pnas.1217470110 PMC3581893 23382235

[10] Gupta YK, Dagdas YF, Martinez-Rocha AL, Kershaw MJ, Littlejohn GR, et al (2015) Septin-Dependent Assembly of the Exocyst Is Essential for Plant Infection by Magnaporthe oryzae. Plant Cell 27: 3277–3289. 10.1105/tpc.15.00552 26566920 PMC4682301

[11] Lambeth JD (2004) NOX enzymes and the biology of reactive oxygen. Nat Rev Immunol 4: 181–189. 15039755 10.1038/nri1312

[12] Sumimoto H (2008) Structure, regulation and evolution of Nox-family NADPH oxidases that produce reactive oxygen species. FEBS Journal 275: 3249–3277. 10.1111/j.1742-4658.2008.06488.x 18513324

[13] Scott B (2015) Conservation of fungal and animal NADPH oxidase complexes. Molecular Microbiology: n/a-n/a. 10.1111/mmi.12946 25620385

[14] Aguirre J, Rios-Momberg M, Hewitt D, Hansberg W (2005) Reactive oxygen species and development in microbial eukaryotes. Trends Microbiol 13: 111–118. 15737729 10.1016/j.tim.2005.01.007

[15] Takemoto D, Tanaka A, Scott B (2007) NADPH oxidases in fungi: diverse roles of reactive oxygen species in fungal cellular differentiation. Fungal Genet Biol 44: 1065–1076. 17560148 10.1016/j.fgb.2007.04.011

[16] Heller J, Tudzynski P (2011) Reactive oxygen species in phytopathogenic fungi: signaling, development, and disease. Annu Rev Phytopathol 49: 369–390. 10.1146/annurev-phyto-072910-095355 21568704

[17] Takemoto D, Kamakura S, Saikia S, Becker Y, Wrenn R, et al (2011) Polarity proteins Bem1 and Cdc24 are components of the filamentous fungal NADPH oxidase complex. Proc Natl Acad Sci U S A 108: 2861–2866. 10.1073/pnas.1017309108 21282602 PMC3041104

[18] Lara-Ortiz T, Riveros-Rosas H, Aguirre J (2003) Reactive oxygen species generated by microbial NADPH oxidase NoxA regulate sexual development in Aspergillus nidulans. Molecular Microbiology 50: 1241–1255. 14622412 10.1046/j.1365-2958.2003.03800.x

[19] Malagnac F, Lalucque H, Lepere G, Silar P (2004) Two NADPH oxidase isoforms are required for sexual reproduction and ascospore germination in the filamentous fungus Podospora anserina. Fungal Genetics and Biology 41: 982–997. 15465387 10.1016/j.fgb.2004.07.008

[20] Cano-Dominguez N, Alvarez-Delfin K, Hansberg W, Aguirre J (2008) NADPH oxidases NOX-1 and NOX-2 require the regulatory subunit NOR-1 to control cell differentiation and growth in Neurospora crassa. Eukaryot Cell 7: 1352–1361. 10.1128/EC.00137-08 18567788 PMC2519770

[21] Giesbert S, Schurg T, Scheele S, Tudzynski P (2008) The NADPH oxidase Cpnox1 is required for full pathogenicity of the ergot fungus Claviceps purpurea. Molecular Plant Pathology 9: 317–327. 10.1111/j.1364-3703.2008.00466.x 18705873 PMC6640299

[22] Segmuller N, Kokkelink L, Giesbert S, Odinius D, van Kan J, et al (2008) NADPH Oxidases are involved in differentiation and pathogenicity in Botrytis cinerea. Molecular Plant-Microbe Interactions 21: 808–819. 10.1094/MPMI-21-6-0808 18624644

[23] Schürmann J, Buttermann D, Herrmann A, Giesbert S, Tudzynski P (2013) Molecular Characterization of the NADPH Oxidase Complex in the Ergot Fungus Claviceps purpurea: CpNox2 and CpPls1 Are Important for a Balanced Host-Pathogen Interaction. Molecular Plant-Microbe Interactions 26: 1151–1164. 10.1094/MPMI-03-13-0064-R 23777432

[24] Scott B, Eaton CJ (2008) Role of reactive oxygen species in fungal cellular differentiations. Curr Opin Microbiol 11: 488–493. 10.1016/j.mib.2008.10.008 18983937

[25] Tudzynski P, Heller J, Siegmund U (2012) Reactive oxygen species generation in fungal development and pathogenesis. Curr Opin Microbiol 15: 653–659. 10.1016/j.mib.2012.10.002 23123514

[26] Lacaze I, Lalucque H, Siegmund U, Silar P, Brun S (2014) Identification of NoxD/Pro41 as the homologue of the p22 NADPH oxidase subunit in fungi. Mol Microbiol. 10.1111/mmi.12876 25424886

[27] Siegmund U, Marschall R, Tudzynski P (2015) BcNoxD, a putative ER protein, is a new component of the NADPH oxidase complex in Botrytis cinerea. Molecular Microbiology 95: 988–1005. 10.1111/mmi.12869 25402961

[28] Lambou K, Malagnac F, Barbisan C, Tharreau D, Lebrun MH, et al (2008) The Crucial Role of the Pls1 Tetraspanin during Ascospore Germination in Podospora anserina Provides an Example of the Convergent Evolution of Morphogenetic Processes in Fungal Plant Pathogens and Saprobes. Eukaryotic Cell 7: 1809–1818. 10.1128/EC.00149-08 18757568 PMC2568061

[29] Siegmund U, Heller J, van Kann JAL, Tudzynski P (2013) The NADPH Oxidase Complexes in *Botrytis cinerea*: Evidence for a Close Association with the ER and the Tetraspanin Pls1. PLoS ONE 8: e55879 10.1371/journal.pone.0055879 23418468 PMC3572182

[30] Lee YJ, Yang Z (2008) Tip growth: signaling in the apical dome. Current Opinion in Plant Biology 11: 662–671. 10.1016/j.pbi.2008.10.002 18977167 PMC2613292

[31] Foreman J, Demidchik V, Bothwell JHF, Mylona P, Miedema H, et al (2003) Reactive oxygen species produced by NADPH oxidase regulate plant cell growth. Nature 422: 442–446. 12660786 10.1038/nature01485

[32] Cardenas L (2009) New findings in the mechanisms regulating polar growth in root hair cells. Plant Signaling & Behavior 4: 4–8. 19568333 10.4161/psb.4.1.7341 PMC2634060

[33] Jackson SL, Heath IB (1993) Roles of calcium ions in hyphal tip growth. Microbiological Reviews 57: 367–382. 8336672 10.1128/mr.57.2.367-382.1993 PMC372914

[34] Klosterman SJ, Subbarao KV, Kang S, Veronese P, Gold SE, et al (2011) Comparative Genomics Yields Insights into Niche Adaptation of Plant Vascular Wilt Pathogens. Plos Pathogens 7: e1002137 10.1371/journal.ppat.1002137 21829347 PMC3145793

[35] Wang S, Xing H, Hua C, Guo H-s, Zhang J (2016) An improved single-step cloning strategy simplifies the Agrobacterium tumefaciens-mediated transformation (ATMT)-based gene disruption method in Verticillium dahliae. Phytopathology. 10.1094/PHYTO-10-15-0280-R 26780432

[36] Gao F, Zhou BJ, Li GY, Jia PS, Li H, et al (2010) A glutamic acid-rich protein identified in *Verticillium dahliae* from an insertional mutagenesis affects microsclerotial formation and pathogenicity. PLoS One 5: e15319 10.1371/journal.pone.0015319 21151869 PMC2998422

[37] Bourett TM, Howard RJ (1990) In vitro development of penetration structures in the rice blast fungus Magnaporthe grisea. Canadian Journal of Botany 68: 329–342.

[38] Nair R, Raina S, Keshavarz T, Kerrigan MJP (2011) Application of fluorescent indicators to analyse intracellular calcium and morphology in filamentous fungi. Fungal Biology 115: 326–334. 10.1016/j.funbio.2010.12.012 21530914

[39] Thewes S (2014) Calcineurin-Crz1 Signaling in Lower Eukaryotes. Eukaryotic Cell 13: 694–705. 10.1128/EC.00038-14 24681686 PMC4054267

[40] Liu S, Hou Y, Liu W, Lu C, Wang W, et al (2015) Components of the Calcium-Calcineurin Signaling Pathway in Fungal Cells and Their Potential as Antifungal Targets. Eukaryotic Cell 14: 324–334. 10.1128/EC.00271-14 25636321 PMC4385803

[41] Xiong D, Wang Y, Tang C, Fang Y, Zou J, et al (2015) VdCrz1 is involved in microsclerotia formation and required for full virulence in Verticillium dahliae. Fungal Genetics and Biology 82: 201–212. 10.1016/j.fgb.2015.07.011 26235044

[42] Choi J, Kim Y, Kim S, Park J, Lee Y-H (2009) MoCRZ1, a gene encoding a calcineurin-responsive transcription factor, regulates fungal growth and pathogenicity of Magnaporthe oryzae. Fungal Genetics and Biology 46: 243–254. 10.1016/j.fgb.2008.11.010 19111943

[43] Soriani FM, Malavazi I, da Silva Ferreira ME, Savoldi M, Von Zeska Kress MR, et al (2008) Functional characterization of the Aspergillus fumigatus CRZ1 homologue, CrzA. Molecular Microbiology 67: 1274–1291. 10.1111/j.1365-2958.2008.06122.x 18298443

[44] Kim S, Hu J, Oh Y, Park J, Choi J, et al (2010) Combining ChIP-chip and Expression Profiling to Model the MoCRZ1 Mediated Circuit for Ca(2+)/Calcineurin Signaling in the Rice Blast Fungus. Plos Pathogens 6: e1000909 10.1371/journal.ppat.1000909 20502632 PMC2873923

[45] Zoller M (2009) Tetraspanins: push and pull in suppressing and promoting metastasis. Nat Rev Cancer 9: 40–55. 10.1038/nrc2543 19078974

[46] Moribe H, Konakawa R, Koga D, Ushiki T, Nakamura K, et al (2012) Tetraspanin Is Required for Generation of Reactive Oxygen Species by the Dual Oxidase System in *Caenorhabditis elegans* . PLoS Genet 8: e1002957 10.1371/journal.pgen.1002957 23028364 PMC3447965

[47] Ibi M, Katsuyama M, Fan C, Iwata K, Nishinaka T, et al (2006) NOX1/NADPH oxidase negatively regulates nerve growth factor-induced neurite outgrowth. Free Radical Biology and Medicine 40: 1785–1795. 16678016 10.1016/j.freeradbiomed.2006.01.009

[48] Yang Z (2008) Cell Polarity Signaling in Arabidopsis. Annual Review of Cell and Developmental Biology 24: 551–575. 10.1146/annurev.cellbio.23.090506.123233 18837672 PMC2739732

[49] Semighini CP, Harris SD (2008) Regulation of apical dominance in Aspergillus nidulans hyphae by reactive oxygen species. Genetics 179: 1919–1932. 10.1534/genetics.108.089318 18689883 PMC2516069

[50] Clergeot PH, Gourgues M, Cots J, Laurans F, Latorse MP, et al (2001) PLS1, a gene encoding a tetraspanin-like protein, is required for penetration of rice leaf by the fungal pathogen Magnaporthe grisea. Proceedings of the National Academy of Sciences of the United States of America 98: 6963–6968. 11391010 10.1073/pnas.111132998 PMC34461

[51] Gourgues M, Brunet-Simon A, Lebrun MH, Levis C (2004) The tetraspanin BcPls1 is required for appressorium-mediated penetration of Botrytis cinerea into host plant leaves. Molecular Microbiology 51: 619–629. 14731267 10.1046/j.1365-2958.2003.03866.x

[52] Veneault-Fourrey C, Parisot D, Gourgues M, Lauge R, Lebrun MH, et al (2005) The tetraspanin gene ClPLS1 is essential for appressorium-mediated penetration of the fungal pathogen Colletotrichum findemuthianum. Fungal Genetics and Biology 42: 306–318. 15749050 10.1016/j.fgb.2005.01.009

[53] Sheppard FR, Kelher MR, Moore EE, McLaughlin NJD, Banerjee A, et al (2005) Structural organization of the neutrophil NADPH oxidase: phosphorylation and translocation during priming and activation. Journal of Leukocyte Biology 78: 1025–1042. 16204621 10.1189/jlb.0804442

[54] Ginsel L, Onderwater J, Fransen J, Verhoeven A, Roos D (1990) Localization of the low-Mr subunit of cytochrome b558 in human blood phagocytes by immunoelectron microscopy. Blood 76: 2105–2116. 2173636

[55] Cross A, Jones O, Harper AM, Segal AW (1981) Oxidation-reduction properties of the cytochrome b found in the plasma-membrane fraction of human neutrophils. A possible oxidase in the respiratory burst. Biochem j 194: 599–606. 7306004 10.1042/bj1940599 PMC1162784

[56] Calafat J, Kuijpers T, Janssen H, Borregaard N, Verhoeven A, et al (1993) Evidence for small intracellular vesicles in human blood phagocytes containing cytochrome b558 and the adhesion molecule CD11b/CD18. Blood 81: 3122–3129. 8098969

[57] DeLeo FR, Renee J, McCormick S, Nakamura M, Apicella M, et al (1998) Neutrophils exposed to bacterial lipopolysaccharide upregulate NADPH oxidase assembly. Journal of Clinical Investigation 101: 455 9435318 10.1172/JCI949 PMC508585

[58] Potocký M, Jones MA, Bezvoda R, Smirnoff N, Žárský V (2007) Reactive oxygen species produced by NADPH oxidase are involved in pollen tube growth. New Phytologist 174: 742–751. 17504458 10.1111/j.1469-8137.2007.02042.x

[59] Xiang Y, Huang X, Wang T, Zhang Y, Liu Q, et al (2007) ACTIN BINDING PROTEIN29 from Lilium pollen plays an important role in dynamic actin remodeling. Plant Cell 19: 1930–1946. 17586658 10.1105/tpc.106.048413 PMC1955736

[60] Carroll AD, Moyen C, Van Kesteren P, Tooke F, Battey NH, et al (1998) Ca2+, annexins, and GTP modulate exocytosis from maize root cap protoplasts. Plant Cell 10: 1267–1276. 9707528 10.1105/tpc.10.8.1267 PMC144062

[61] Lee YJ, Szumlanski A, Nielsen E, Yang Z (2008) Rho-GTPase–dependent filamentous actin dynamics coordinate vesicle targeting and exocytosis during tip growth. J Cell Biol 181: 1155–1168. 10.1083/jcb.200801086 18591430 PMC2442199

[62] Sambrook J (2001) Molecular cloning: a laboratory manual / Sambrook Joseph, David W. Russell; Russell DW, Cold Spring Harbor L, editors. Cold Spring Harbor, N.Y: Cold Spring Harbor Laboratory.

