# Supplementary figures and images for: Hyphopodium-Specific VdNoxB/VdPls1-Dependent ROS-Ca2+ Signaling Is Required for Plant Infection by Verticillium dahliae
Source: PLoS Pathog. 2016 Jul 27;12(7):e1005793. doi: 10.1371/journal.ppat.1005793 (PMC4962994; doi:10.1371/journal.ppat.1005793)

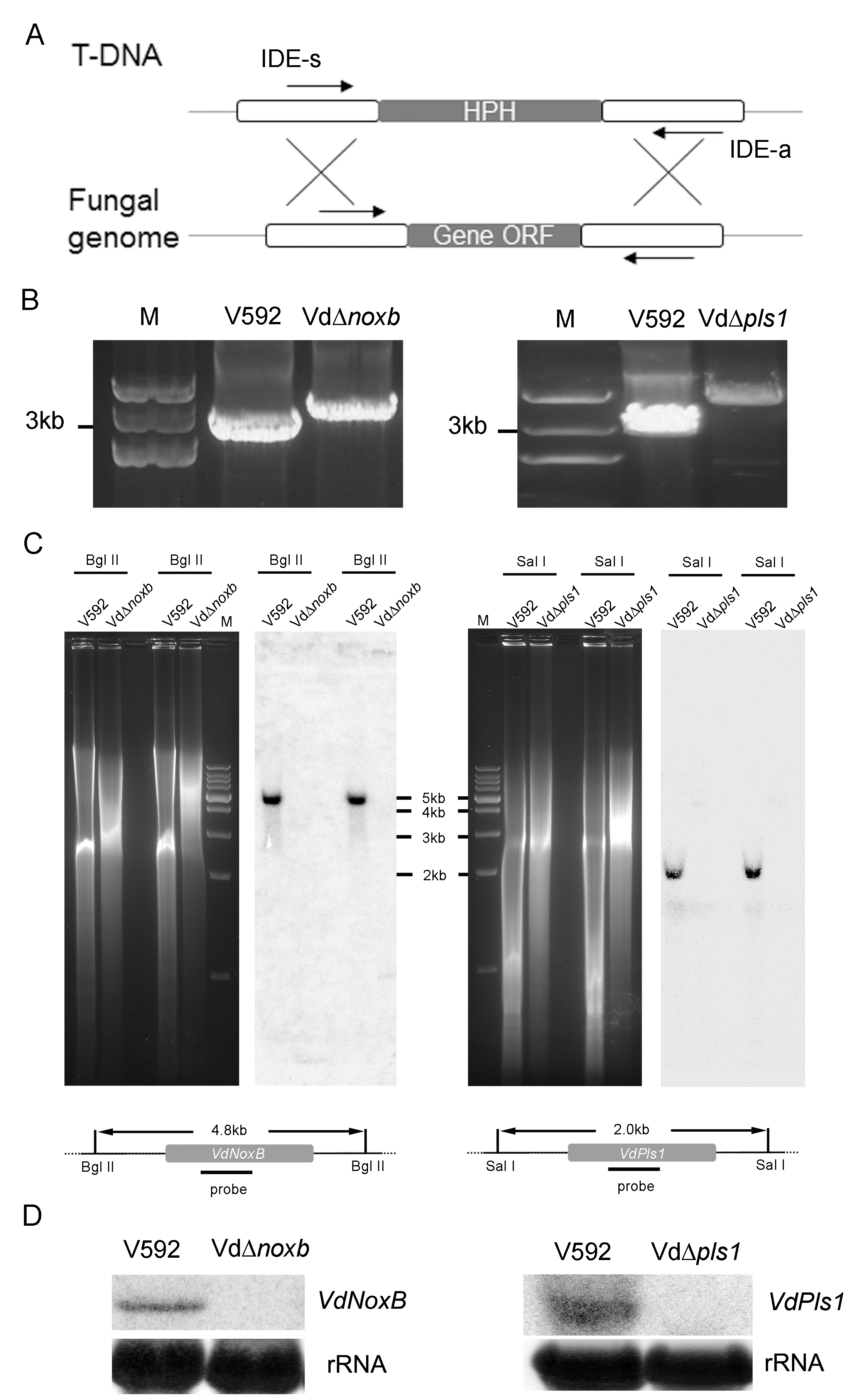

Supplement: S1 Fig — A. Schematic representation of the homologous recombination event involved in the targeted replacement of VdNoxB and VdPls1. B. PCR identification of the knockout mutant with the primers IDE-s and IDE-a indicated in A. C. Southern blot analysis of targeted gene deletion mutants. Bgl II digested genomic DNA from V592 wild type strain and two putative Δnoxb transformants were gel fractionated (left) and blotted (right) with the probe indicated in the schematic diagram. Sal I digested genomic DNA from V592 wild type strain and two putative Δpls1 transformants were analyzed as described above. D. Northern blot analysis of the expression of VdNoxB and VdPls1 in knockout mutants with the probe used for southern blot analysis. (TIF) [file ppat.1005793.s001.tif]

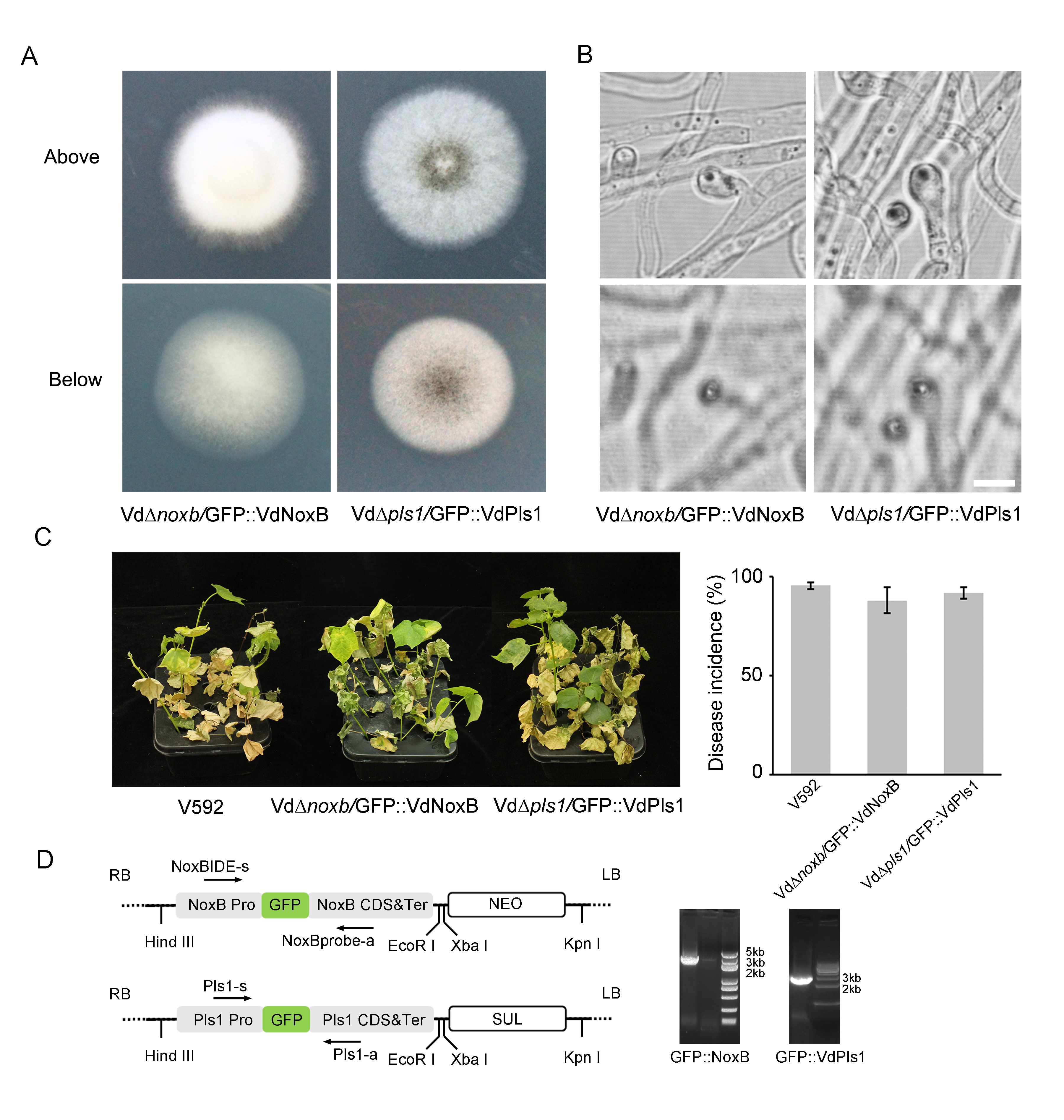

Supplement: S2 Fig — A. Penetration assay of complemented strains of VdΔnoxb/GFP::VdNoxB and VdΔpls1/VdGFP::Pls1. ‘Above’ and ‘Below’ show colonies grown on cellophane membrane and medium after the membrane was removed, respectively. B. Observation of penetration peg development on the cellophane membrane at 2 dpi. Penetration pegs (the dark pin) were observed from the hyphopodia, the second row was focused at 5 μm below the first row. Bar = 5 μm. C. Restoration of pathogenicity of VdΔnoxb/GFP::VdNoxB and VdΔpls1/GFP::VdPls1 on cotton plants. Photographs were taken at 30 dpi. D. Schematic representation of the GFP::VdNoxB and GFP::VdPls1 constructs. PCR confirmation of transformants with the primers indicated in schematic diagram. (TIF) [file ppat.1005793.s002.tif]

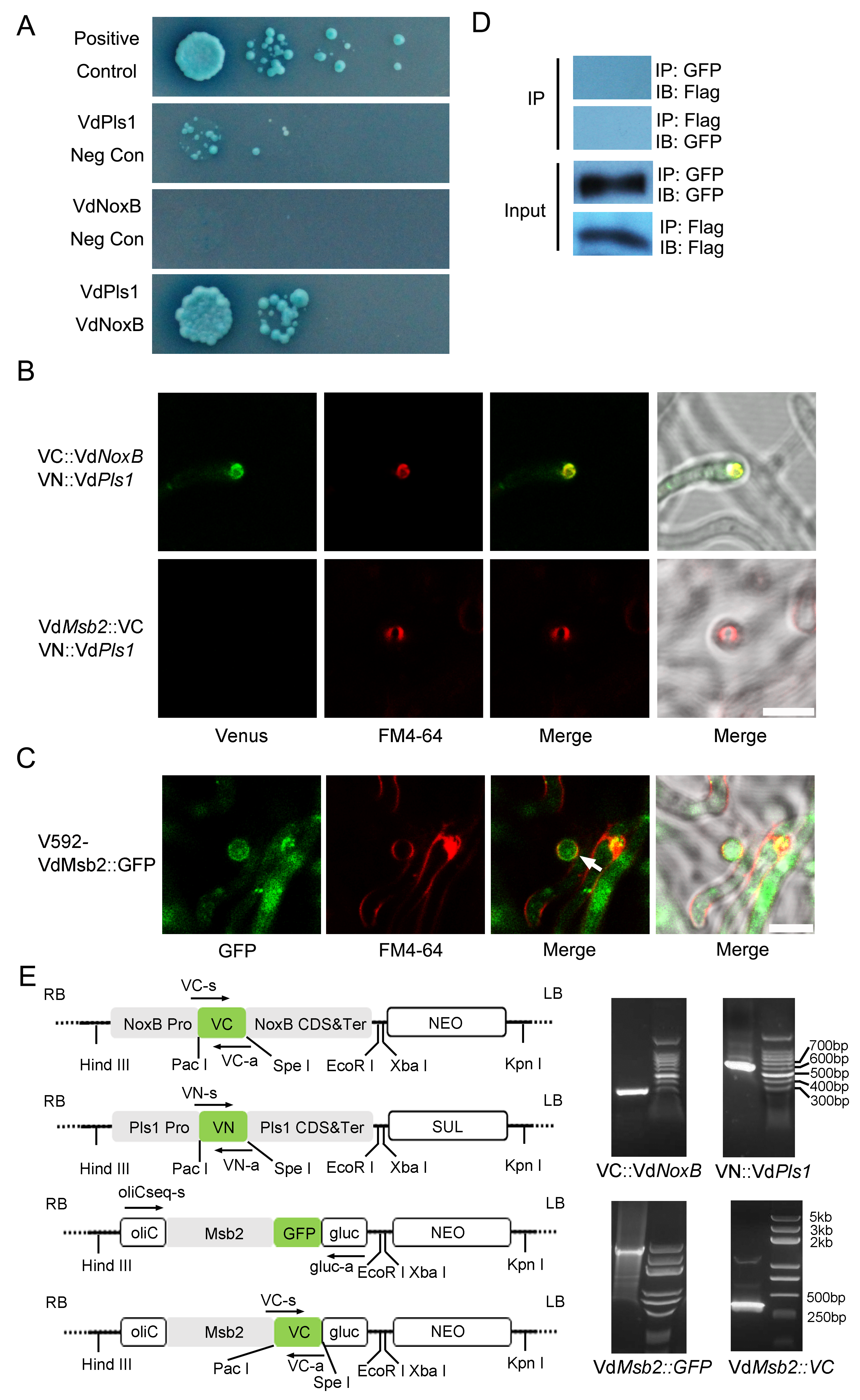

Supplement: S3 Fig — A. Yeast two-hybrid assay showing direct interaction of VdNoxB and VdPls1. Constructs of pPR3N-NoxB, pBT3N-Pls1 and respective control vector were co-transformed in Yeast NMY51 and cultured on the SD-Leu-Trp-His-Ade/10 mM 3AT/X-Gal medium with 1, 1:10, 1:100, and 1:1000 dilutions. Positive control: pBT3N-Pls1/pOST1-NubI. Negative control: pBT3N-Pls1/pPR3-N and pPR3N-NoxB/pBT3N-AR. B. BiFC assay showing interaction of VdNoxB and VdPls1 in vivo. VN-VdNoxB and VC-VdPls1 were transformed in V592 and Venus signal on the membrane of penetration peg was detected. Plasma membrane was stained with FM4-64. VdMsb2::VC was co-transformed with VN::VdPls1 as the negative control. Bar = 5μm. C. Localization of VdMsb2::GFP. Localization of VdMsb2 in V592-Msb2::GFP strain incubated on cellophane membrane. The image was taken at 2 dpi and shows that VdMsb2 localized on the membrane of fungal hyphae and aggregated with the membrane of penetration peg (indicated by an arrow). Bar = 5μm. D. Immunoblot of the failed co-immunoprecipitation result. V592 expressing GFP::VdNoxB and 3Flag::VdPls1 was grown on MM medium overlaid with cellophane for 2 days to conduct Co-IP assay. E. Schematic representation of the BiFC assay related constructs. PCR confirmation of positive transformants with the primers indicated in schematic diagram. (TIF) [file ppat.1005793.s003.tif]

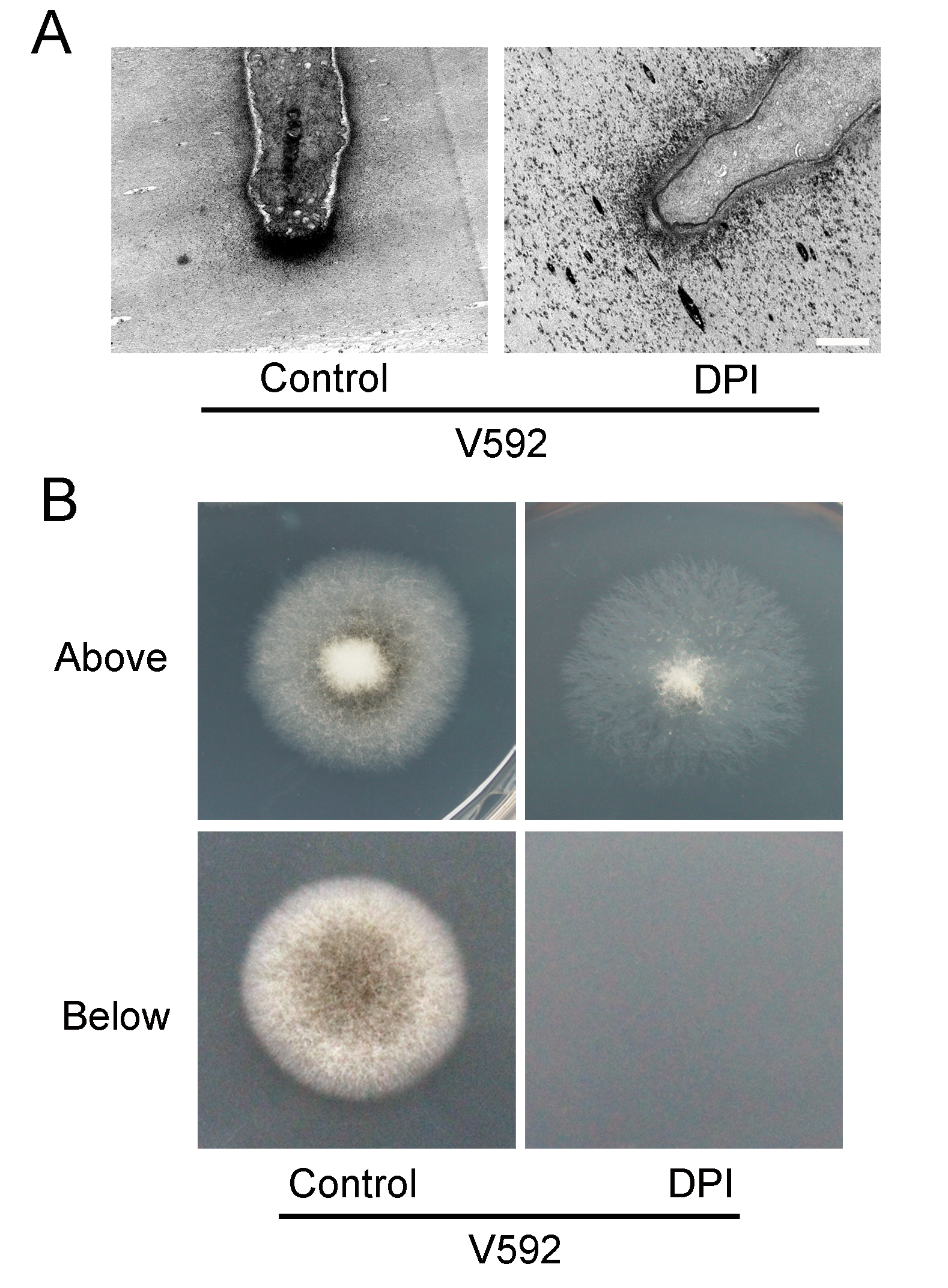

Supplement: S4 Fig — A. Detection of cerium deposits by TEM. V592 was treated with DPI 2 hours before cerium chloride staining, and the control was treated with DMSO. Cerium deposits were detected on the membrane at the tip of penetration peg in V592 treated with DMSO. DPI prevented ROS accumulation at the apex of penetration peg. Bar = 0.5 μm. B. V592 penetration assay with DPI treatment. V592 was grown on cellophane for 1 day to develop hyphopodia followed by transfer of cellophane to MM medium containing DPI for another 2 days. MM medium containing DMSO was used as a control. The image shows growth of a V592 colony on MM medium with a cellophane layer (above) and removal of the cellophane membrane (below). Photographs in the first row were taken at 7 dpi. V592 penetration of cellophane was blocked on MM medium containing DPI. (TIF) [file ppat.1005793.s004.tif]

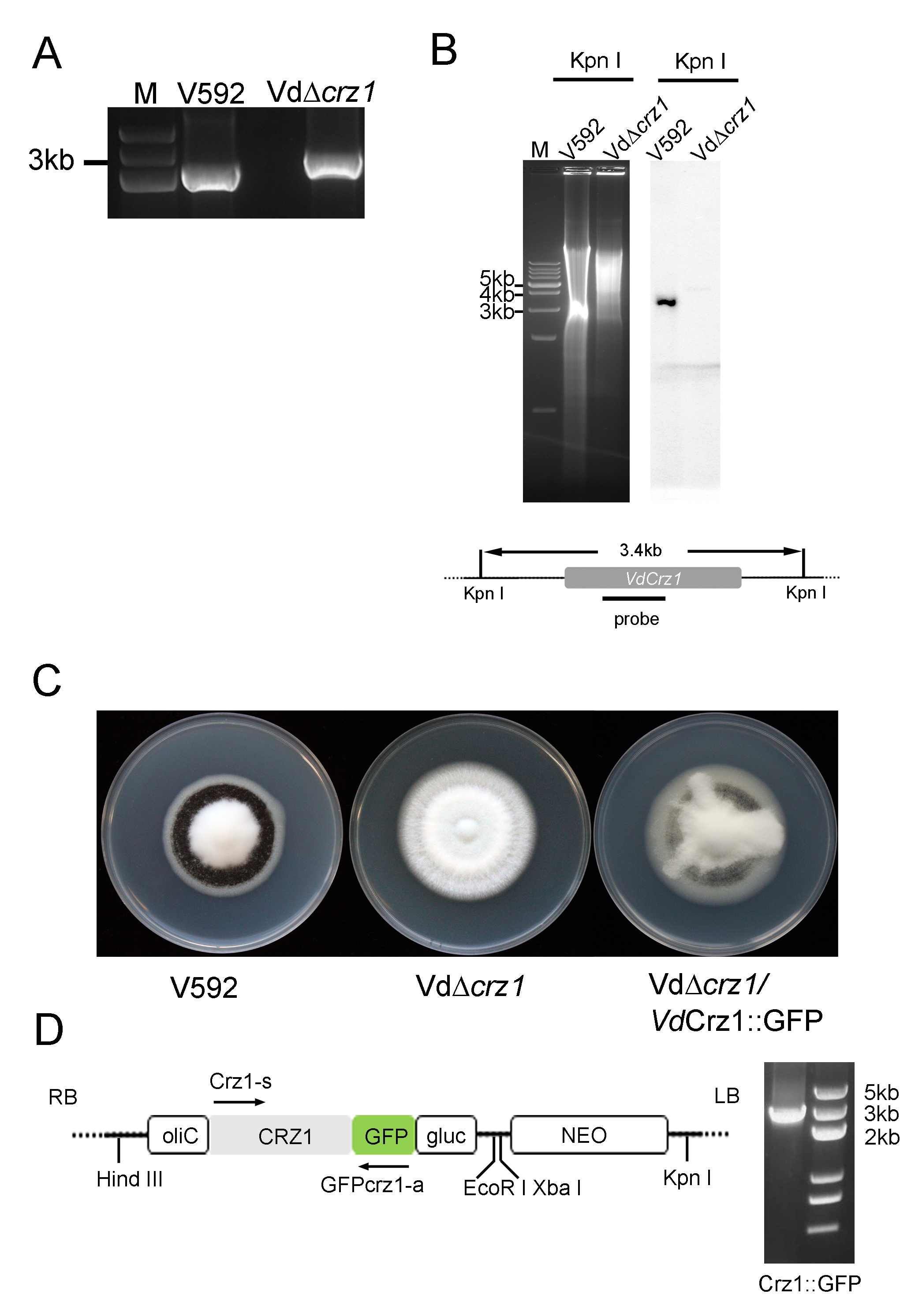

Supplement: S5 Fig — A. PCR identification of the VdCrz1 knockout mutant with primers Crz1IDE-s and Crz1IDE-a as indicated in S1A Fig. B. Southern blot analysis of VdCrz1 deletion mutant. Kpn I digested genomic DNA from V592 wild type strain and putative Δcrz1 transformant were gel fractionated (left) and blotted (right) with the probe indicated in the schematic diagram. C. Colony morphology of V592, VdΔcrz1 and VdΔcrz1/VdCrz1::GFP on PDA plates after 2 weeks post-incubation. D. Schematic representation of the VdCrz1::GFP construct. PCR confirmation of transformant with the primers indicated in schematic diagram. (TIF) [file ppat.1005793.s005.tif]
