# Supplementary material for: Hyphopodium-Specific VdNoxB/VdPls1-Dependent ROS-Ca2+ Signaling Is Required for Plant Infection by Verticillium dahliae
Source: PLoS Pathog. 2016 Jul 27;12(7):e1005793. doi: 10.1371/journal.ppat.1005793 (PMC4962994; doi:10.1371/journal.ppat.1005793)
Supplement: S1 Table — (DOCX) [file ppat.1005793.s006.docx]

**S1 Table *Verticillium dahliae* strains used in this study.**

| Strain | Genotype description | Reference |
| --- | --- | --- |
| V592 | Wild type | Gao and Zhou et al., 2010 |
| Vd*∆noxb* | *VdNoxB* deletion mutant | This study |
| Vd*∆pls1* | *VdPls1* deletion mutant | This study |
| Vd*∆noxb/VdNoxB* | *VdNoxB* complementary strain | This study |
| Vd*∆pls1/VdPls1* | *VdPls1* complementary strain | This study |
| V592-GFP | Transformant of V592 expressing GFP | Zhao et al., 2014 |
| Vd*∆noxb*-GFP | Transformant of Vd*∆noxb* expressing GFP | This study |
| Vd*∆pls1*-GFP | Transformant of Vd*∆pls1* expressing GFP | This study |
| Vd*∆noxb/VdNoxB*-GFP | Transformant of *Vd∆noxb/VdNoxB* expressing GFP | This study |
| Vd*∆pls1/VdPls1*-GFP | Transformant of *Vd∆pls1/VdPls1*  expressing GFP | This study |
| Vd*∆noxb*/GFP::VdNoxB | Complemented strain of Vd*∆noxb* expressing GFP::VdNoxB | This study |
| Vd*∆pls1*/GFP::VdPls1 | Complemented strain of Vd*∆pls1* expressing GFP::VdPls1 | This study |
| VC::VdNoxB/VN::VdPls1 | Transformant of V592 expressing VC::VdNoxB and VN::VdPls1 | This study |
| VdMsb2::VC/ VN::VdPls1 | Transformant of V592 expressing VdMsb2::VC and VN::VdPls1 | This study |
| V592-VdMsb2::GFP | Transformant of V592 expressing VdMsb2::GFP | This study |
| V592-GFP::VdNoxB | Transformant of V592 expressing GFP::VdNoxB | This study |
| Vd*∆pls1*-GFP::VdNoxB | *VdPls1* deletion mutant of V592-GFP::VdNoxB | This study |
| V592-GFP::VdNoxB/3Flag::VdPls1 | Transformant of V592 expressing GFP::VdNoxB and 3Flag::VdPls1 | This study |
| Vd*∆crz1* | *VdCrz1* deletion mutant | This study |
| Vd*∆crz1/*VdCrz1::GFP | Complemented strain of Vd*∆crz1* expressing GFP::VdCrz1 | This study |
| V592-VdCrz1::GFP | Transformant of V592 expressing GFP::VdCrz1 | This study |
| Vd*∆noxb*-VdCrz1::GFP | Transformant of Vd∆noxb expressing GFP::VdCrz1 | This study |
| Vd*∆pls1*--VdCrz1::GFP | Transformant of Vd∆pls1 expressing GFP::VdCrz1 | This study |
| Vd*∆noxb/VdNoxB*-VdCrz1::GFP | Transformant of Vd∆noxb/VdNoxB expressing GFP::VdCrz1 | This study |
| Vd*∆pls1/VdPls1*-VdCrz1::GFP | Transformant of Vd∆pls1/VdPls1 expressing GFP::VdCrz1 | This study |
