# Supplementary material for: Hyphopodium-Specific VdNoxB/VdPls1-Dependent ROS-Ca2+ Signaling Is Required for Plant Infection by Verticillium dahliae
Source: PLoS Pathog. 2016 Jul 27;12(7):e1005793. doi: 10.1371/journal.ppat.1005793 (PMC4962994; doi:10.1371/journal.ppat.1005793)
Supplement: S2 Table — (DOCX) [file ppat.1005793.s007.docx]

**S2 Table Primers used in this study**

| Primer | Sequence(5’ →3’) | Application |
| --- | --- | --- |
| KONoxBup-s | gggtttaaucaacaagagccgcaatcaa | *VdNoxB* deletion |
| KONoxBup-a | ggacttaaucgtgctggtagtgctataatcgt |  |
| KONoxBdn-s | ggcattaauagggggcaaggcgtggagcaatt |  |
| KONoxBdn-a | ggtcttaaugaagactcgggcttgatgg |  |
| KOPls1up-s | gggtttaaugctgtgccttccctccgc | *VdPls1* deletion |
| KOPls1up-a | ggacttaaugttgctggcggtctcgagg |  |
| KOPls1dn-s | ggcattaauacgaccgtaaagccgatctcc |  |
| KOPls1dn-a | ggtcttaaugctgtacccgctggtgttctg |  |
| KOCrz1up-s | gggtttaautgaggcgatggatgggctac | *VdCrz1* deletion |
| KOCrz1up-a | ggacttaautgaggctgctgttggtggc |  |
| KOCrz1dn-s | ggcattaaucgggtacgggattcggtgac |  |
| KOCrz1dn-a | ggtcttaaucgagaagacgacaagggagcag |  |
| NoxBIDE-s | aagctccctccttcttcatcc | Deletion identification |
| NoxBIDE-a | aaaatcctcttccaaccctcg |  |
| Pls1IDE-s | gcacttccaaggatgagggatgaga |  |
| Pls1IDE-a | gaagaagaggcaaggaaatg |  |
| Crz1IDE-s | cgcctcgctttgtcttgtc |  |
| Crz1IDE-a | gaatgcgttgtgcctctgtc |  |
| NoxBprobe-s | acaaccttggcgtctatggc |  |
| NoxBprobe-a | gctggggtgctgaatgactt |  |
| Pls1probe-s | tggtcgccgatgtgcttt |  |
| Pls1probe-a | actgggtggtgagggtcagg |  |
| Crz1probe-s | ccagggcacgagtccttcca |  |
| Crz1probe-s | agagcgtcggcacgagcaaa |  |
| NoxBup-s | acgacggccagtgccaagcttctccaggtaacagggaaga | VdNoxB complementation and GFP::VdNoxB fusion |
| NoxBup-a | accatgttaattaacgtgctggtagtgctataa |  |
| GFPnoxb-s | gcacgttaattaacatggtgagcaagggcgag |  |
| GFPnoxb-a | cctccactagtgccgcctccgcccttgtacagctcgtccatg |  |
| NoxBdn-s | gcggcactagtggaggcggaggaatggactactactcgatgg |  |
| NoxBdn-a | ggctgtctagaactagaattcacagggtgaggcagaaact |  |
| Pls1up-s | acgacggccagtgccaagcttgggcacggcacaaagggtca | VdPls1 complementation and GFP::VdPls1 fusion |
| Pls1up-a | accatgttaattaagttgctggcggtctcgaggact |  |
| GFPpls1-s | gcaacttaattaacatggtgagcaagggcgag |  |
| GFPpls1-a | cctccactagtgccgcctccgcccttgtacagctcgtccatg |  |
| Pls1dn-s | gcggcactagtggaggcggaggaatggtcaacaagatcctcgcga |  |
| Pls1dn-a | ggccgctctagaactagaattcgaagaagaggcaaggaaatg |  |
| Pls1com-a | ggctgtctagaactagaattcgaagaagaggcaaggaaatg |  |
| Pls1-s | gcacttccaaggatgagggatgaga |  |
| Pls1-a | tgccgaagacagcagtgaagat |  |
| NoxBpn-s | gtatcaacgcagagtggccattacggccatggactactactcgatgg | Y2H |
| NoxBpn-a | atcgaattctcgagaggccgaggcggccctagaagttctccttgccccatttg |  |
| Pls1bn-s | atcgaattcctgcagggccattacggccatggtcaacaagatcctcgcga |  |
| Pls1bn-a | agctacttaccatggggccgaggcggccctagaggctgcggtatccgctct |  |
| ARbn-s | atcgaattcctgcagggccattacggccatgtcgtcgccatgcgtgtgt |  |
| ARbn-a | agctacttaccatggggccgaggcggcctcagtagacggaacactcgagc |  |
| VN-s | gttaattaacatggactacaaagacgatgacgacaaggtgagcaagggcgag | VN::VdPls1 fusion |
| VN-a | tcactagtgccgcctccgccctcgatgttgtggcggatct |  |
| VC-s | gttaattaacatgtacccatacgacgtaccagattacgctgacaagcagaagaacggca | VC::VdNoxB fusion |
| VC-a | tcactagtgccgcctccgcccttgtacagctcgtcca |  |
| Neo-s | ctctagacagccgccttcgcaagcgct | pNEO construction |
| Neo-a | cggtaccggccagcagtagacacttgg |  |
| oliC-s | acgacggccagtgccaagctttgcagctgtggagccgcattccc | VdMsb2::GFP fusion |
| oliC-a | cttgacctgcatttggatcgattgtgatgtgatgg |  |
| Msb2-s | cgatccaaatgcaggtcaagtccgtcctt |  |
| Msb2-a | tccgttaattaagccgcctccgccattccagccaagagagttc |  |
| GFPmsb2-s | ggcttaattaacggaggcggaggaatggtgagcaagggcgag |  |
| GFPmsb2-a | acatacgctaactagtttacttgtacagctcgtccatg |  |
| gluc-s | gtaaactagttagcgtatgtagataagatgtatg |  |
| gluc-a | ggctgtctagaactagaattcgatcttgttggggggaaggg |  |
| oliCseq-s | cccatcatccatctcctcaccc |  |
| VCmsb2-s | ggcttaattaacggaggcggaggagacaagcagaagaacggca | VdMsb2::VC fusion |
| VCmsb2-a | aaactagtttaagcgtaatctggtacgtcgtatgggtacttgtacagctcgtccat |  |
| 3Flag-s | gagaccgccagcaacttaattaacatggtcgactacaaagaccatgatg | 3Flag::VdPls1 fusion |
| 3Flag-a | cattcctccgcctccactagtcttatcgtcatcgtcctt |  |
| oliCcrz1-s | acgacggccagtgccaagctttgcagctgtggagccgcattccc | VdCrz1::GFP fussion |
| oliCcrz1-a | gttaattaattggatcgattgtgatgtga |  |
| Crz1-s | tcacaatcgatccaattaattaac atggatcagcaagctcaacatcg |  |
| Crz1-a | tcctccgcctccgttaattaagccgcctccgcc gcgaccgccatagtcgctcgcat |  |
| GFPcrz1-s | ttaattaacggaggcggagga atggtgagcaagggcgaggag |  |
| GFPcrz1-a | tatctacatacgctaggatccttacttgtacagctcgtcca |  |
| gluccrz1-s | ggatcctagcgtatgtagataagat |  |
| gluccrz1-a | ggccgctctagaactagaattcgatcttgttggggggaaggggtt |  |
| Crz1-s | tttactcgcgcgtacaacct | qRT-PCR |
| Crz1-a | aacttcttctcgcccgagtg |  |
| Lcc-s | tacctcttccactgccacatc |  |
| Lcc-a | gtttccgccagcattacca |  |
| Mde-s | gctggtagtgacggttccaa |  |
| Mde-a | cagcaccaaaagtgtgaccg |  |
| MFS-s | ccgaagatctcccagcagtc |  |
| MFS-a | acccgtctctatcacctgct |  |
| Rhom-s | cccatgattggcccctcttt |  |
| Rhom-a | caccaaatgggctgttgagc |  |
| Elf-s | ccattgatatcgcactgtgg |  |
| Elf-a | tggagataccagcctcgaac |  |
